# Knocking Down *CDKN2A* in 3D hiPSC-Derived Brown Adipose Progenitors Potentiates Differentiation, Oxidative Metabolism and Browning Process

**DOI:** 10.3390/cells12060870

**Published:** 2023-03-10

**Authors:** Yasmina Kahoul, Xi Yao, Frédérik Oger, Maeva Moreno, Souhila Amanzougarene, Mehdi Derhourhi, Emmanuelle Durand, Raphael Boutry, Amélie Bonnefond, Philippe Froguel, Christian Dani, Jean-Sébastien Annicotte, Christophe Breton

**Affiliations:** 1 Univ. Lille, INSERM, CNRS, CHU Lille, Institut Pasteur de Lille, U1283-UMR8199-EGID, F-59000 Lille, France; 2 Faculté de Médecine, CNRS, INSERM, iBV, Université Côte d’Azur, CEDEX 2, F-06107 Nice, France; 3 Department of Metabolism, Imperial College London, London SW7 2BX, UK; 4 Univ. Lille, Inserm, CHU Lille, Institut Pasteur Lille, U1167 - RID-AGE - Facteurs de Risque et Déterminants Moléculaires des Maladies liées au Vieillissement, F-59000 Lille, France

**Keywords:** human induced pluripotent stem cells, adipocytes, brown adipose progenitor, browning, 3D culture, *CDKN2A*, UCP1, thermogenesis

## Abstract

Human induced pluripotent stem cells (hiPSCs) have the potential to be differentiated into any cell type, making them a relevant tool for therapeutic purposes such as cell-based therapies. In particular, they show great promise for obesity treatment as they represent an unlimited source of brown/beige adipose progenitors (hiPSC-BAPs). However, the low brown/beige adipocyte differentiation potential in 2D cultures represents a strong limitation for clinical use. In adipose tissue, besides its cell cycle regulator functions, the cyclin-dependent kinase inhibitor 2A (*CDKN2A*) locus modulates the commitment of stem cells to the brown-like type fate, mature adipocyte energy metabolism and the browning of adipose tissue. Here, using a new method of hiPSC-BAPs 3D culture, via the formation of an organoid-like structure, we silenced *CDKN2A* expression during hiPSC-BAP adipogenic differentiation and observed that knocking down *CDKN2A* potentiates adipogenesis, oxidative metabolism and the browning process, resulting in brown-like adipocytes by promoting UCP1 expression and beiging markers. Our results suggest that modulating *CDKN2A* levels could be relevant for hiPSC-BAPs cell-based therapies.

## 1. Introduction

Obesity is considered the main risk factor for type 2 diabetes (T2D), mainly due to the excessive accumulation of adipose tissue (AT) [1]. The expansion of AT in obese individuals is a direct cause of the comorbidities, due to the excessive accumulation of triglycerides (TG) within adipocytes, leading to inflammation and insulin resistance. In mammals, there are two major types of AT that are anatomically and functionally distinct: white (WAT) and brown (BAT). White adipocytes store excess energy as TG and release free fatty acids as energy substrate during periods of negative energy balance. BAT differs from WAT by its cellular origin, and is specialized in energy expenditure and the production of heat, mainly through active fat oxidation [2]. Elevated energy expenditure in BAT is correlated with high expression levels of a specific mitochondrial protein named uncoupling protein 1 (UCP1). More recently, the presence of a subtype of thermogenic adipocytes within WAT that also expresses UCP1 has been reported. These inducible adipocytes, named beige, are distinct from white and brown adipocytes. They mainly arise from noradrenergic stimulation or cold exposure. The conversion of white adipocytes into brown-like adipocytes is called browning [3]. Obese individuals are characterized by increased WAT mass and decreased brown and beige AT mass and activity [4]. Increasing energy expenditure by BAT activation or by promoting the browning of WAT may represent a new therapeutic avenue to prevent insulin resistance in obesity and T2D [5]. In humans, cold exposure enhances metabolic activity and thermogenesis in BAT. This increase is accompanied by increased insulin sensitivity in diabetic patients [6]. The transplantation of BAT or brown adipocytes isolated from human adipose progenitors (APs) into the visceral cavity of mice reverses metabolic syndrome and T2D, constituting a potential translatable therapeutic tool to improve metabolic health [7]. Since beige adipocytes can arise through de novo differentiation from undifferentiated APs or via the conversion of mature white adipocytes into UCP1-positive cells, referred to as transdifferentiation [8,9,10], the identification of selective molecular pathways and underlying mechanisms involved in beige adipocyte biogenesis may represent a first step towards innovative therapeutic options.

Genome-wide association studies have established that several single nucleotide polymorphisms, including loss-of function mutations in the cyclin-dependent kinase inhibitor 2A (*CDKN2A*) locus, affect glycemia, insulin values and T2D risk, implying a role in glucose and insulin sensitivity regulation [11,12]. The human *CDKN2A* locus encodes two proteins, the Cyclin Dependent Kinase inhibitory (CDKI) p16INK4a protein and the p53 regulatory protein p14ARF (p19ARF in mice). The p16INK4a protein is a potent CDKI preventing the binding of CDK4/6 to Cyclin D, controlling the CDK4-pRB-E2F1 pathway; whereas p14ARF mainly exerts its activity via the inhibition of MDM2, a ubiquitin-ligase that promotes the degradation of p53 [11,12]. In AT, besides its cell cycle regulator functions (i.e., anti-proliferative and tumor suppressor), the *CDKN2A* locus also controls the commitment of stem cells to the brown-like type fate and mature adipocyte energy metabolism [13,14,15]. We have shown that mice with a germline disruption of the *Cdkn2a* gene (*Cdkn2a*^−/−^) fed a high-fat diet are protected against diet induced obesity (DIO) by increasing thermogenesis via inguinal (ing) WAT beiging, resulting in improved insulin sensitivity associated with the activation of the PKA pathway [16]. In this study, we have also observed that *CDKN2A* expression is increased in adipocytes from obese, compared to lean, subjects [16]. Consistent with these findings, a recent study reported that silencing *Cdkn2a* expression in cold-inducible beige APs results in a rejuvenation of beige adipocyte formation, restoring cold-induced thermogenesis in old mice [13]. The authors also showed that silencing *Cdkn2a* expression in UCP1+ cells within ingWAT that display progenitor-like characteristics stimulates new beige fat formation through cell proliferation via a cell-autonomous role [17]. Overall, these data indicate the existence of an inverse correlation between the expression level of *CDKN2A* and beige adipocyte activity, further supporting the notion that cell-cycle genes may be involved in controlling a white-to-beige/brown fat transition that involves APs, beige adipocyte expansion and their activity in a cell-autonomous manner.

Human induced pluripotent stem cells (hiPSCs) have the potential to be differentiated into any cell type, making these cells an unlimited source for studying cell-based therapy. In particular, several studies have established the therapeutic potential of hiPSCs differentiated into brown adipocytes progenitors (hiPSC-BAP) against obesity and associated metabolic disorders [18]. However, the limitation of the use of hiPSC-BAPs in 2D cultures is due to their low adipocyte capacity and their low expression levels of UCP1 [18]. Here, to overcome this limit, we report a new method of 3D culture, via the formation of an organoid-like structure, which enhances the capacity for differentiation and the browning process of hiPSC-BAPs [19]. We have previously reported that silencing *CDKN2A* expression during hiPSC-BAP adipogenic differentiation in 2D cultures promotes UCP1 expression [16]. In this study, we investigated the effects of *CDKN2A* silencing in hiPSC-BAP in improved 3D cultures. Understanding how *Cdkn2a* can relay to initiate a thermogenic program in hiPSC-BAPs is a first step to envisage activating beiging as a new putative therapy to alleviate the effects of obesity and to prevent insulin resistance and T2D. RNA-sequencing (RNA-seq) analysis and kinase activity profiling of hiPSC-BAP further demonstrate that *CDKN2A* silencing enhances pathways involved in adipogenesis, oxidative metabolism and the browning process, resulting in the reprogramming of brown-like adipocytes by promoting UCP1 expression and beiging markers.

## 2. Materials and Methods

### 2.1. Cell Culture and siRNA Experiments

hiPSC-BAPs, derived from the hiPSC line NOK6 were grown in standard tissue culture conditions at 37 °C with 5% CO_2_ as previously described [20]. The growth medium was DMEM low glucose supplemented with L-glutamine (2 mM), penicillin–streptomycin 5000 IU/mL–5000 g/mL (Pen/Strep), 10% FBS and 2.5 ng/mL FGF2. 

#### 2.1.1. Generation of hiPSC-Derived Brown-like Adipospheres

The generation of spheroids and their adipogenic differentiation was performed as we recently described in detail [21]. Briefly, 1 × 10^6^ hiPSC-BAPs were seeded per well of 24 well Ultra-Low Attachment (ULA) plate (Corning 3473, Fischer scientific, Illkirch-Graffenstaden, France) for three days for spheroid formation. Then, to differentiate spheroids, the growth medium was changed to a differentiation medium composed of EBM-2 (Lonza, Colmar, France) supplemented with 0.1% FCS, IBMX (0.5 mM), dexamethasone (0.25 μM), T3 (0.2 nM), insulin (170 nM), rosiglitazone (1 μM), SB431542 (5 μM), and a EGM-2 cocktail (Lonza, CC-3121) including ascorbic acid, hy-drocortisone and EGF. IBMX and dexamethasone were maintained only for the first 3 days of differentiation. SB431542 and EGF were removed after the first 9 days of differentiation. Spheroids were maintained in the differentiation medium for up to 20 days, with the medium changed once a week. 

#### 2.1.2. siRNA Transfection

siRNAs (Human *CDKN2A* siRNASMART pool, GEHealth Bio-Sciences, Rosersberg, Sweden) were transfected at the time when hiPSC-BAPs were in suspension for spheroid formation. One hundred nM siRNAs were transfected in a medium containing 60% DMEM low glucose, 40% MCDB-201, 1× ITS, dexamethasone (10^−9^ M), and ascorbic sodium acid (100 mM) using HiPerFect (Qiagen, Courtaboeuf, France) transfection reagent as described by the supplier. Cells were then maintained in conditions to form spheroids and were induced to differentiate as described above.

### 2.2. RNA Extraction and RNA-Sequencing

Total RNA was extracted from the hiPSC-BAP 3D at D0 and D10 of differentiation using TRIzolTM Reagent (Sigma-Aldrich, Saint-Quentin-Fallavier, France). The quality of the RNAs was verified with RNA 6000 nanochips on the agilent 2100 bioanalyser. Purified RNA (200 ng) was used for the library preparation. Briefly, RNA libraries were prepared using the TruSeq Stranded mRNA Library Preparation Kit (Illumina, San Diego, CA, USA) following the manufacturer’s instructions. The libraries were sequenced on the NextSeq system (Illumina) using a paired-end 2x75 bp protocol. Three biological replicates per condition were sequenced. The GEO accession number for the sequencing data was GSE223241.

### 2.3. Proteins Extraction and PamGene Kinase Assay 

Proteins from spheroids and adipospheres were extracted for PamGene kinase assay as previously described [16]. Tyrosine (PTK) and serine/threonine kinase (STK) activity was investigated with PTK and STK microarrays purchased from PamGene (PamGene International BV, ’s-Hertogenbosch, The Netherlands). The experiments were performed as described in the manufacturer’s instructions.

### 2.4. Bioinformatic Analysis

For RNA sequencing, the demultiplexing of the sequence data was performed using bcl2fastq Conversion Software (Illumina; bcl2fastq v2.19.1). The trimming of adapter sequences was performed using cutadapt software (version 1.7.1). Reads quality control was assessed using FastQC (v0.11.5). Subsequently, sequence reads from FASTQ files were aligned to the human genome GRCh38, downloaded from Ensembl 108. Alignment was performed using STAR aligner (version 2.5.2b). Over 19 millions of 75 base pairs PE-reads reads were generated per sample. The normalized counts of the different genes and isoforms were performed using RSEM (v1.2.31) using a GTF from Ensembl 108. Finally differential expression was performed using R version 3.6.3 and DESeq2 package v1.24.0. An adjusted *p*-value < 0.05, Log2FC > 1 and LogFC < −1 were set as thresholds. We then performed pathway analysis using the core analysis function of Ingenuity Pathway analysis (IPA) (Qiagen) and the Gene Set Enrichment Analysis (GSEA) was done using GSEA software version 4.3.2 (GSEA; http://software.broadinstitute.org/gsea/ (accessed on 3 October 2022)). All GSEA data showed had a *p*-value < 0.05.

For Pamgene analysis, image acquisition and data analysis were performed according to the manufacturer’s instructions as previously described [16]. Data and upstream kinase analysis were conducted using the Bionavigator software v.6.3.67.0 developed by PamGene. Peptides and kinases with an adjusted *p*-value < 0.05, LogFC > 1 and LogFC < −1 were set as thresholds.

### 2.5. Statistical Analysis

Data are presented as mean ±SEM. Statistical analyses were performed using unpaired two-tailed Student’s *t*-test, using GraphPad Prism software. Differences were considered statistically significant at *p* < 0.05 (* *p* < 0.05; ** *p* < 0.01 and *** *p* < 0.001).

## 3. Results

### 3.1. Characterization of the Differentiation Process of hiPSC-BAPs into Adipocytes in 3D Culture

**Figure 1:**
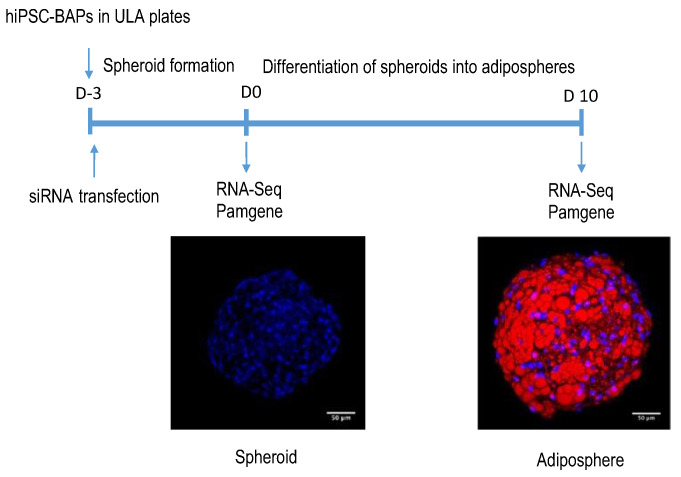
**Protocol of hiPSC-BAP differentiation into adipospheres.** hiPSC-BAPs were plated into low adherent plates (ULA) and transfected with the control siRNA or the *CDKN2A* siRNA. Three days (D-3) after, spheroids formed and then were induced to undergo differentiation into adipospheres. Confoncal images are shown. Blue: DAPI for nuclei staining. Red: Oil Red O for lipid droplets staining. Scale: 50 µm. RNA-Seq and Pamgene analyses were performed at day 0 (D0) and day 10 (D10).

Adipogenic differentiation of hiPSC-BAPs was performed as summarized in Figure 1. Briefly, hiPSC-BAPs were plated, transfected with siRNA, and differentiation was triggered 3 days later, for 10 days. RNA-seq and Pamgene experiments were performed before (at D0) and after (at D10) differentiation.

#### 3.1.1. Transcriptome Analysis of the Adipogenic Differentiation of hiPSC-BAPs in 3D Culture

**Figure 2:**
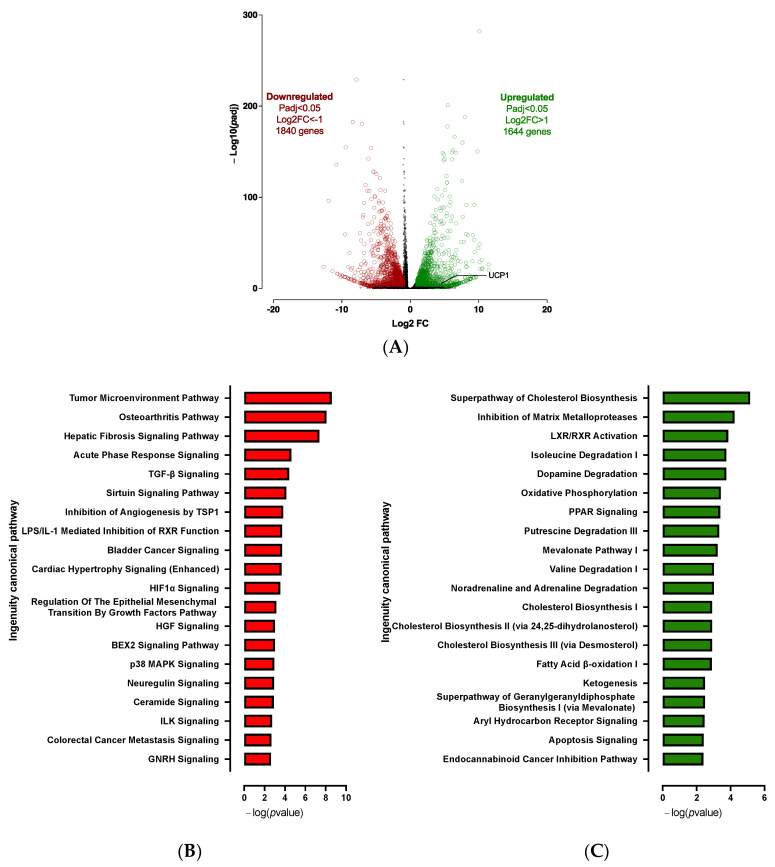
**Transcriptomic analysis of 10 days-differentiated 3D adipospheres (D10 vs. D0).** (**A**) A volcano-plot of differentially regulated *gene* expression. UCP1 is indicated in black. Enrichment of IPA biological process terms for down-regulated (**B**) and up-regulated (**C**) genes following differentiation. IPA terms are plotted against the negative log of corrected *p*-values. Most down-regulated (**B**) and up-regulated (**C**) enriched pathways.

Expression profile differences in the transcriptome of spheroids before differentiation (D0) vs. adipospheres after differentiation (D10) were determined by RNA-seq analysis. The 3D culture markedly affects the hiPSC-BAP mRNA expression levels. Transcriptomic analysis revealed 3484 significantly differentially expressed genes (1644 up-regulated and 1840 down-regulated) between D0 and D10 groups (Figure 2A). Overall, RNA-seq analysis revealed that adipogenesis (i.e., upregulation of PPARγ and CEBPα; downregulation of DIO2), markers of mature adipocytes (i.e., upregulation of ADIPOQ and PLIN1), oxidative metabolism pathways and browning adipocyte capacity (i.e., upregulation of FABP4, CIDEA, PGC1α and UCP1) are markedly activated in 10 days-differentiated 3D adipospheres (Figure S1). We also found that mRNA expression levels of DIO2, an enzyme that catalyzes T4 to T3 conversion [22], were markedly downregulated. Given that T3 is present in the differentiation medium of hiPSC-BAPs, the reduction of DIO2 may reflect active adipocyte differentiation which already adopts a brown-like phenotype [23].

After IPA and GSEA analysis, we found that several pro-adipogenic pathways were markedly modified in the D10 vs. D0 groups. We observed the activation of the cholesterol biosynthesis, LXR/RXR and PPAR signaling pathways (Figure 2C and Figure S3); and the repression of the sirtuin, matrix metalloprotease, acute phase response, osteoarthritis, hepatic fibrosis and TGFβ signaling pathways (Figure 2B and Figure S3). Rosiglitazone, CEBPs, IL4 and Vascular Endothelial Growth Factor (VEGF) are major upstream regulators of up-regulated pathways (Figure S2). Other over-expressed pathways were those involved in cellular oxidative metabolism such as oxidative phosphorylation, fatty acid oxidation, ketogenesis, as well as amino acid and noradrenaline degradation pathways (Figure 2C and Figure S3) which are essential for adipogenesis and browning adipocyte capacity [24,25].

#### 3.1.2. Kinome Profiling of the Adipogenic Differentiation of hiPSC-BAPs in 3D Culture

Then, we used Pamgene arrays containing serine/threonine (STK) and phosphotyrosine (PTK) peptides that were incubated with protein lysates of hiPSC-BAP adipospheres before (D0) and after differentiation (D10), as previously described [16].

**Figure 3:**
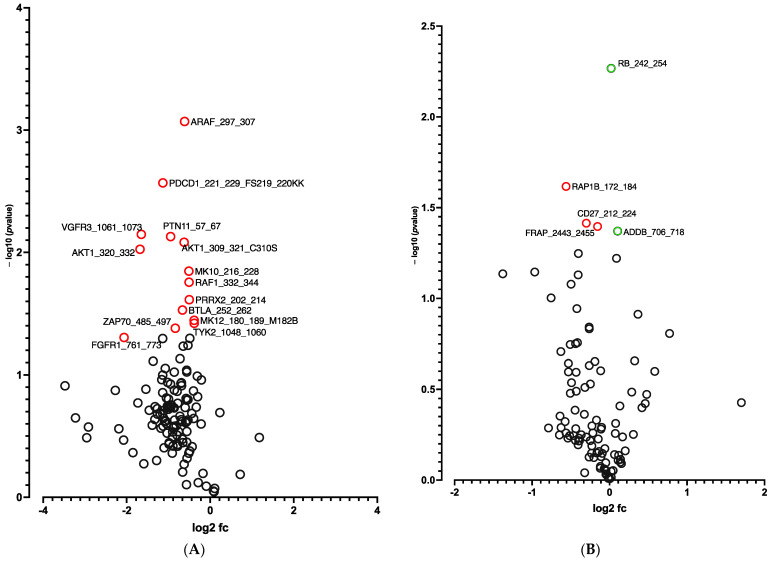

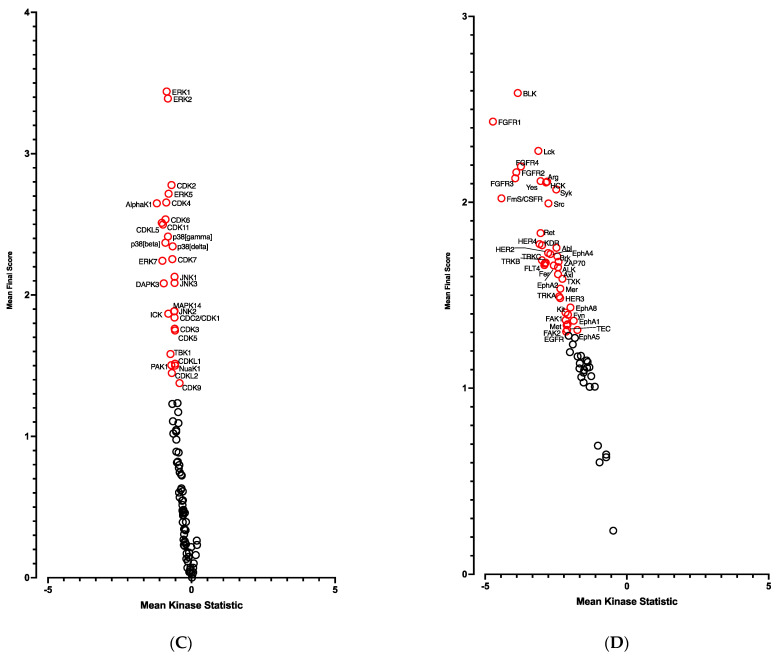
**Kinome analysis in 10 days-differentiated 3D adipospheres (D10 vs. D0).** Volcano-plots of STK (**A**), PTK (**B**)-modulated peptides and STK (**C**), PTK (**D**)-modulated kinases. Green circle: hyperphosphorylated peptide (**A**). Red circle: hypophosphorylated peptide (**A**,**B**) and kinase (**C**,**D**). Dark circle: unmodulated peptide (**A**,**B**) and kinase (**C**,**D**).

A global decrease in phosphorylation of both phosphorylation sites was observed at D10 vs. D0 (Figure 3A,B). Significant differences in phosphorylation for 5 out of 144 peptides (STK, Figure 3A and Table S2) and for 14 out of 196 peptides (PTK, Figure 3B and Table S2) were evidenced. Using a combined Bionavigator and IPA analysis to identify potential upstream kinases, we found several kinases that displayed differential STK (Figure 3C and Table S1) and PTK (Figure 3D and Table S1) phosphorylation at D10 vs. D0. 

**Figure 4:**
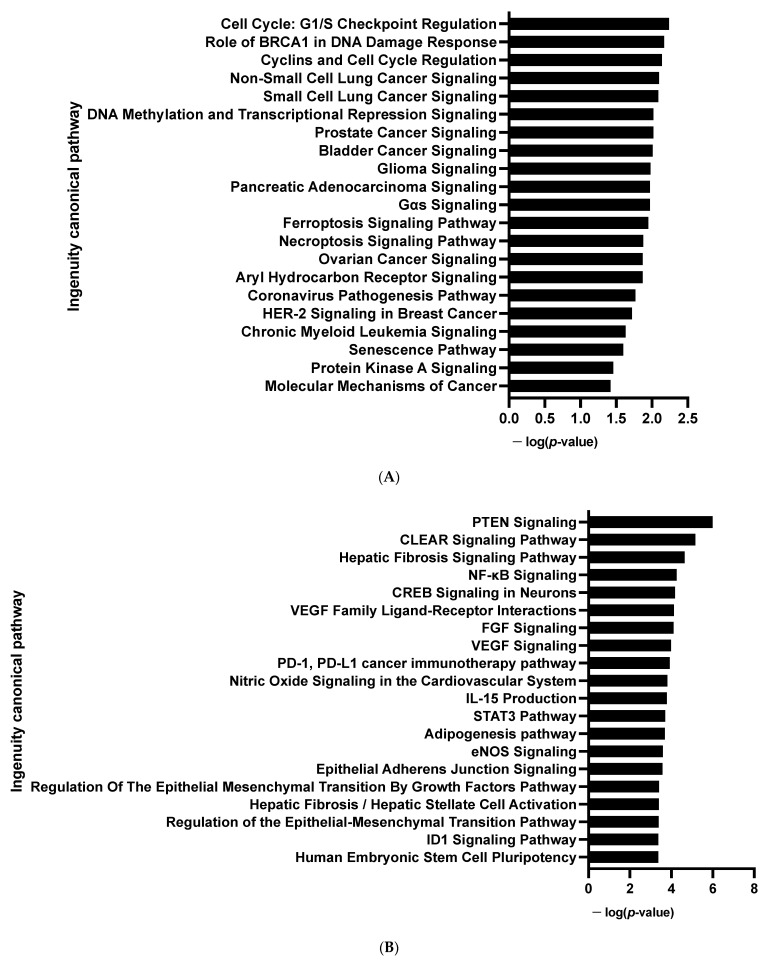
**Enrichment of IPA biological process terms for kinome analysis in 10 days-differentiated 3D adipospheres (D10 vs. D0).** IPA terms are plotted against the negative log of corrected *p*-values. Most modulated enriched pathways in STK (**A**) and in PTK (**B**).

In line with RNA-seq analysis, global pro-adipogenic pathways whose phosphorylation levels were modified were identified, such as cell cycle regulation, adipogenesis, VEGF, fibroblast growth factor (FGF), as well as PTEN and JAK2/STAT3 signaling pathways (STK, Figure 4A and PTK, Figure 4B), which are implicated in proliferation/differentiation during the early stages of adipogenesis [24,25]. Among them, the intracellular mitogen-activated protein kinase (MAPK) and the three pathways: extracellular signal-regulated kinases (ERK1, 2, 5 and 7), Jun N-terminal kinases (JNKs) and p38 (Figure 3C and Table S1), as well as the FGF receptor family (FGFR 1, 2, 3 and 4) (Figure 3D and Table S1), involved in proliferative activity during adipogenesis [24], displayed lower phosphorylation.

### 3.2. Knock-Down of Cdkn2a Potentiates the Capacity of Adipogenic Differentiation of Spheroids at D0

**Figure 5:**
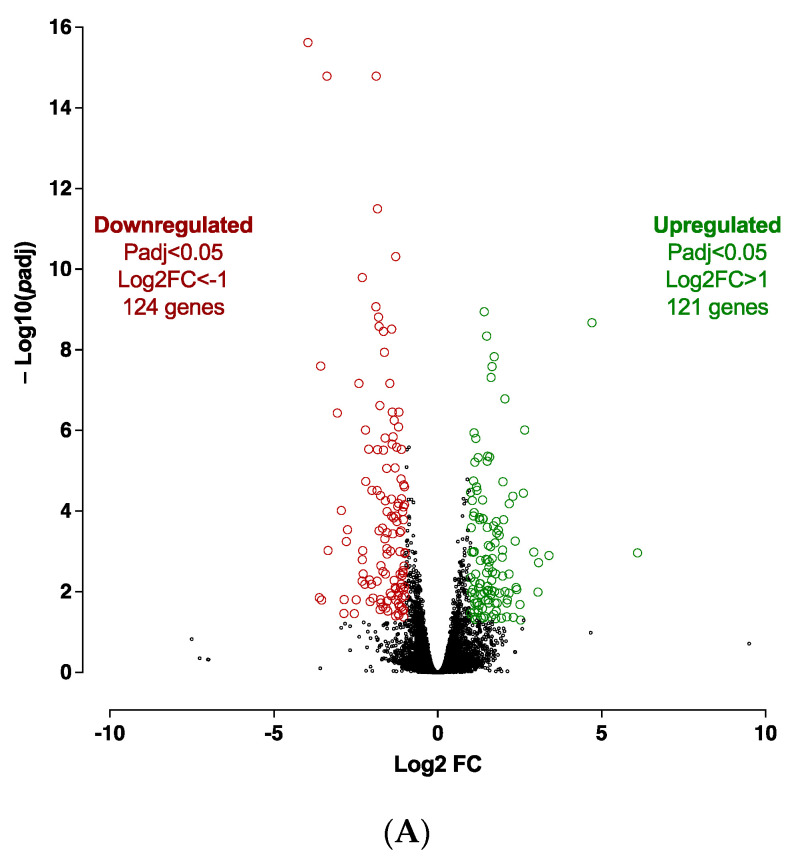

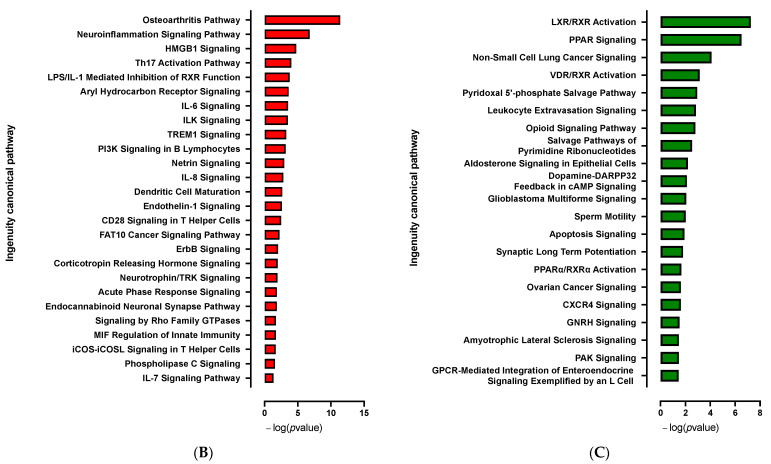
**Transcriptomic analysis at spheroid stages of differentiation (D0) between *CDKN2A*-deficient and control hiPSC-BAPs.** (**A**) A volcano-plot of differentially regulated gene expression. Enrichment of IPA biological process terms for down-regulated (**B**) and up-regulated (**C**) genes of *CDKN2A*-deficient D0 spheroids. IPA terms are plotted against the negative log of corrected *p*-values. Most down-regulated (**B**) and up-regulated (**C**) enriched pathways.

Given that *Cdkn2a* might be required in the APs-specific browning process, we decided to assess selective molecular pathways and the underlying mechanisms involved in this process in *CDKN2A*-deficient hiPSC-BAPs. Expression profile differences in spheroid transcriptome at spheroid stages of differentiation (D0, progenitor stage) between *CDKN2A*-deficient and control spheroids were determined by RNA-seq. Transcriptomic analysis revealed 245 significantly differentially expressed genes (121 up-regulated and 124 down-regulated) between both groups (Figure 5A). The reduction in *CDKN2A* mRNA expression levels was validated in spheroids at D0 (Figure S4). Overall, RNA-seq analysis showed that *CDKN2A*-deficient spheroids exhibit greater adipogenic potential (i.e., downregulation of DIO2) with an anti-inflammatory profile (Figure S4). Computational analysis indicated that pro-adipogenic pathways such as LXR/RXR, PPAR and CXCR4 (chemokine receptor) are activated (Figure 5C and Figure S5); whereas pathways involved in inflammatory response and TGFβ signaling pathways were repressed (Figure 5B and Figure S5) in *CDKN2A*-deficient vs. control spheroids [24]. Pro-adipogenic factors such as PTGER2 (prostaglandin receptor), VEGF, AREG (retinoic acid signaling) andFOXM1 (Forkhead Box M1) are major upstream regulators of up-regulated pathways (Figure S5), and pro-inflammatory signaling pathways (IL6, IL1α, IL17α, NFκB, IL1β, TNF) are major upstream regulators of down-regulated pathways (Figures S5 and S6) [24]. Up-regulation of the molecular pathways involved in oxidative activity was also observed (Figure S6); whereas no significant change was evidenced in UCP1 RNA expression levels at the D0 progenitor stage (Figure S4).

**Figure 6:**
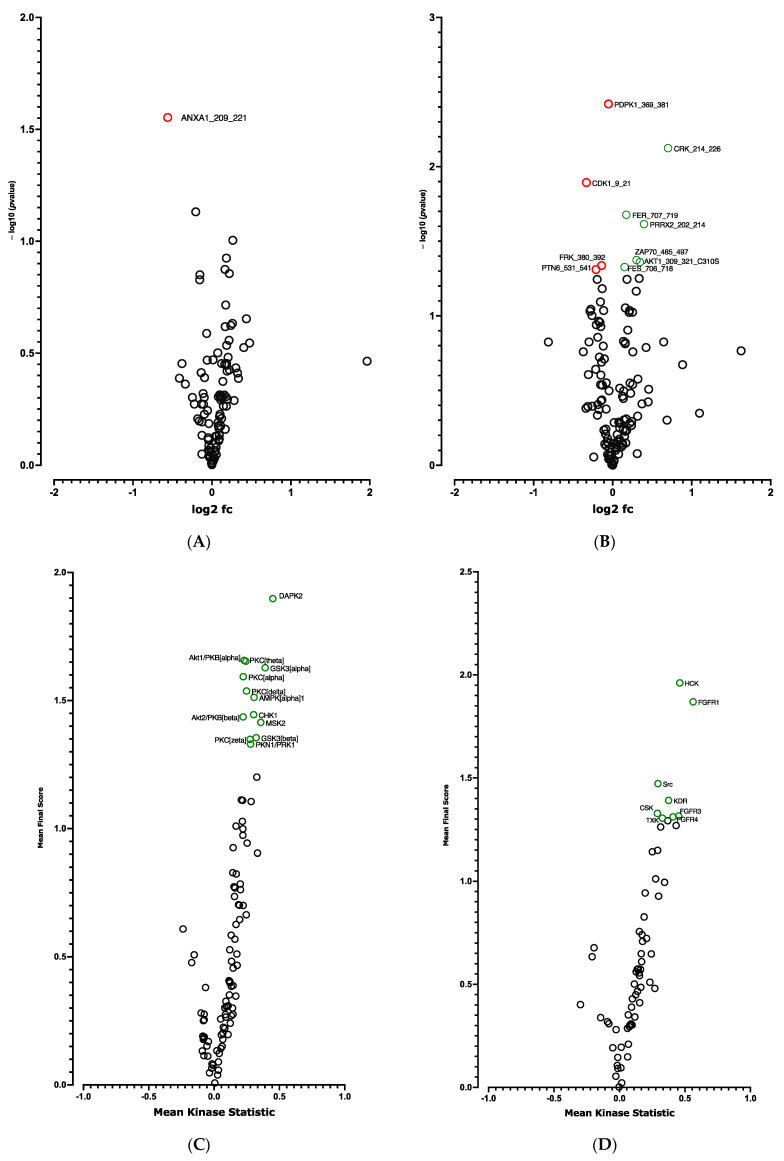
**Kinome analysis at spheroid stages of differentiation (D0) between *CDKN2A*-deficient and control hiPSC-BAPs.** Volcano-plots of STK (A), PTK (**B**)-modulated peptides and STK (**C**), PTK (**D**)-modulated kinases. Green circle: hyperphosphorylated peptide (**B**) and kinase (**C**,**D**). Red circle: hypophosphorylated peptide (**A**,**B**). Dark circle: unmodulated peptide (**A**,**B**) and kinase (**C**,**D**).

**Figure 7:**
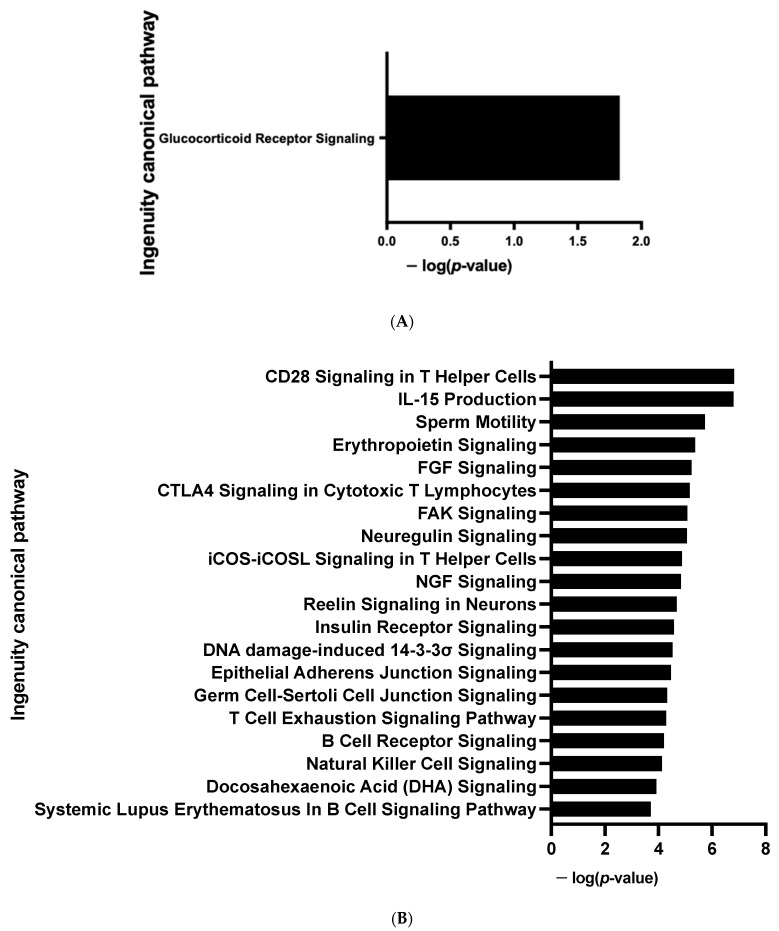
**Enrichment of IPA biological process terms for kinome analysis at spheroid stages of differentiation (D0) between *CDKN2A*-deficient and control hiPSC-BAPs.** IPA terms are plotted against the negative log of corrected *p*-values. Most modulated enriched pathways in STK (**A**) and in PTK (**B**).

The *CDKN2A* products p16INK4a and p19ARF are key regulators of the activity of kinases involved in cell proliferation and senescence [16]. We postulated that modified kinase activity may be involved in the browning process and we performed a global kinome analysis in *CDKN2A*-deficient hiPSC-BAPs. Significant differences in phosphorylation levels for 1 out of 144 peptides (STK, Figure 6A and Table S4) and for 10 out of 196 peptides (PTK, Figure 6B and Table S4) were evidenced between *CDKN2A*-deficient and control spheroids. Using a combined Bionavigator and IPA analysis, we identified several potential modulated signaling pathways and upstream kinases (STK, Figure 6C; PTK, Figure 6D and Table S3). Among them, glucocorticoid (GC) receptor (GR) signaling pathways (STK, Figure 7A), immune pathways (CD28 signaling in T-helper cells, IL15 production, CTA4 signaling in cytotoxic T lymphocyte), as well as FGF, NGF, Focal Adhesion Kinase (FAK) and insulin receptor signaling (PTK, Figure 7B and Table S3), which are implicated in proliferation/differentiation during the early stages of adipogenesis [24], were evidenced.

### 3.3. CDKN2A Invalidation Potentiates Cellular Oxidative Metabolism and Browning Process of Adipospheres at D10

**Figure 8:**
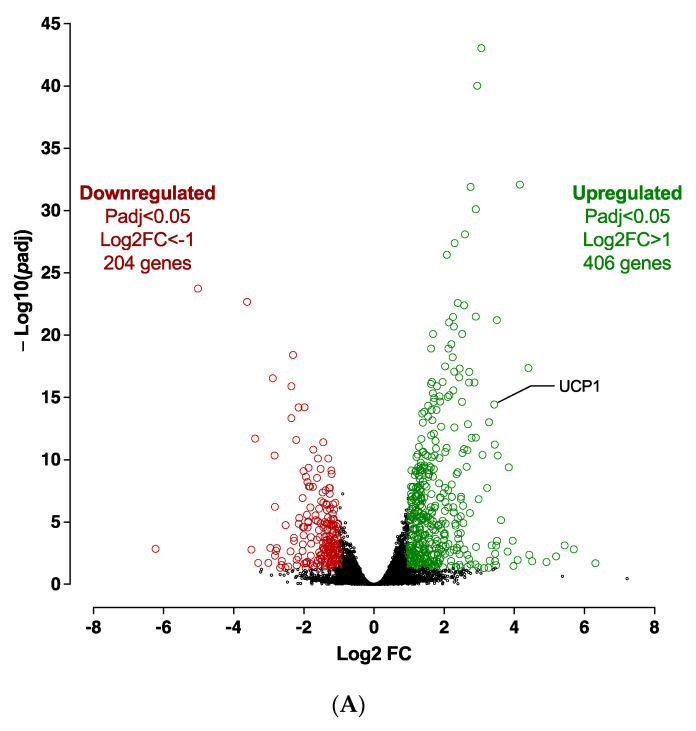

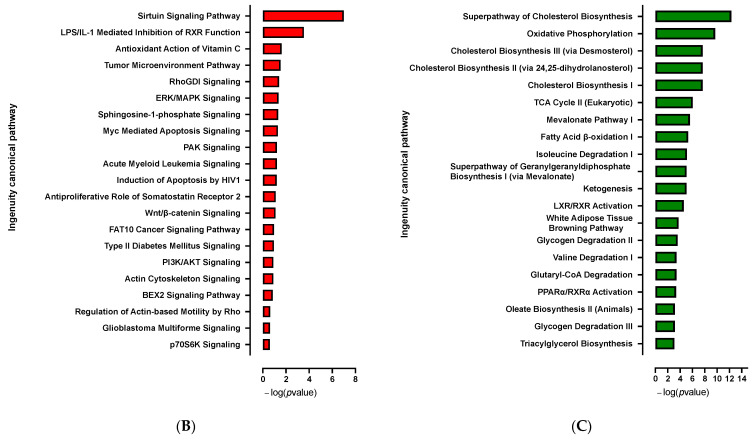
**Transcriptomic analysis in 10 days-differentiated (D10) 3D adipospheres following si*CDKN2A* silencing performed at the progenitor stage 3 days before differentiation.** (**A**) A volcano-plot of differentially regulated gene expression. UCP1 is indicated in black. Enrichment of IPA biological process terms for down-regulated (**B**) and up-regulated (**C**) genes of *CDKN2A*-deficient D10 adipospheres. IPA terms are plotted against the negative log of corrected *p*-values. Most down-regulated (**B**) and up-regulated (**C**) enriched pathways.

The silencing of *CDKN2A* significantly affects the adiposphere mRNA expression levels at D10 (Figure S7). Transcriptomic analysis revealed 610 significantly differentially expressed genes (406 up-regulated and 204 down-regulated) between *CDKN2A*-deficient and control adipospheres (Figure 8A). The reduction of *CDKN2A* mRNA expression levels was validated in spheroids at D10 (Figure S7). Overall, RNA-seq analysis revealed that adipogenesis (i.e., upregulation of PPARγ and CEBPα), markers of mature adipocyte (i.e., upregulation of ADIPOQ and PLIN1), oxidative metabolism and browning process pathways (i.e., upregulation of FABP4, SREBF1, CIDEA, PGC1α, UCP1) are markedly activated in 10 days-differentiated *CDKN2A*-deficient 3D adipospheres (Figure S7).

Computational analysis revealed that, in addition to pro-adipogenic pathways already highlighted at D0, global cellular oxidative metabolism (glycolysis, oxidative phosphorylation, TCA cycle, fatty acid β oxidation, ketogenesis, leucine and valine degradation) and the browning process (white adipose tissue browning pathway, AMPK signaling) are markedly activated [25] (Figure 8C and Figure S9). PPARγ and Sterol Regulatory Element Binding Transcription Factor (SREBF 1 and 2) are major upstream regulators of up-regulated pathways (Figure S8). Several kinase pathways such as p70S6K and PI3K/AKT signaling and sirtuin signaling pathway, which affect the proliferation and differentiation of pre-adipocytes, are repressed [24] (Figure 8B).

**Figure 9:**
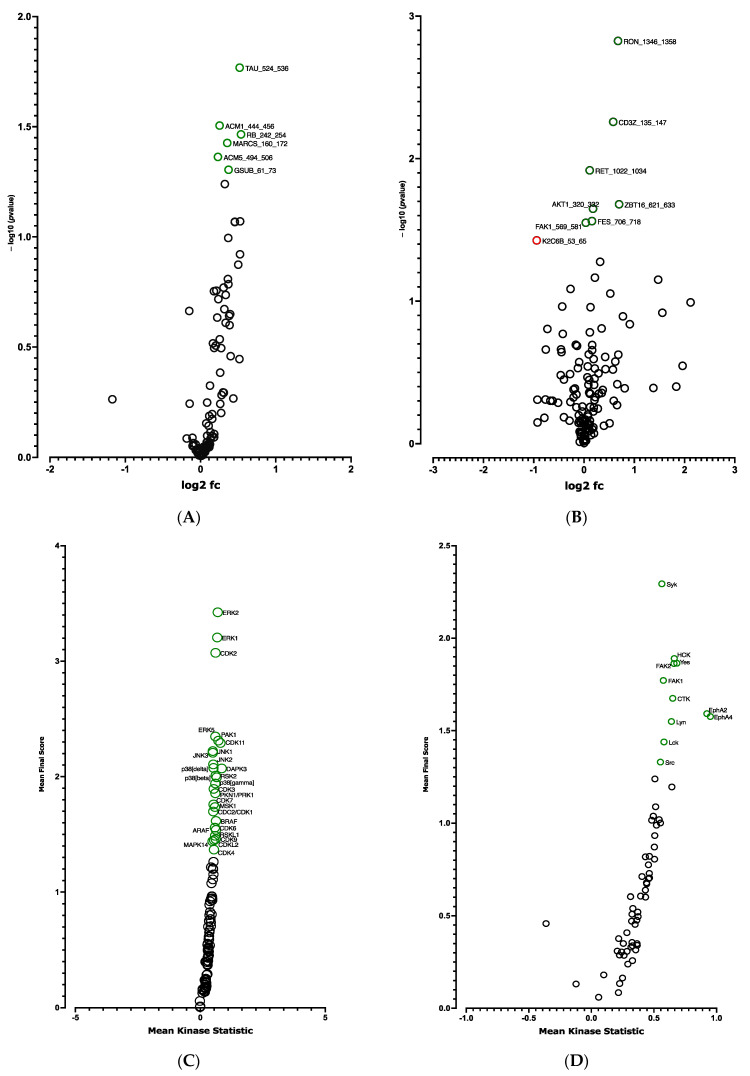
**Kinome analysis in 10 days-differentiated (D10) 3D adipospheres following si*CDKN2A* silencing performed at the progenitor stage 3 days before differentiation**. Volcano-plots illustrating STK (**A**), PTK (B)-modulated peptides and STK (**C**), PTK (**D**)-modulated kinases. Green circle: hyperphosphorylated peptide (**A**,**B**) and kinase (**C**,**D**). Red circle: hypophosphorylated peptide (**B**). Dark circle: unmodulated peptide (**A**,**B**) and kinase (**C**,**D**).

**Figure 10:**
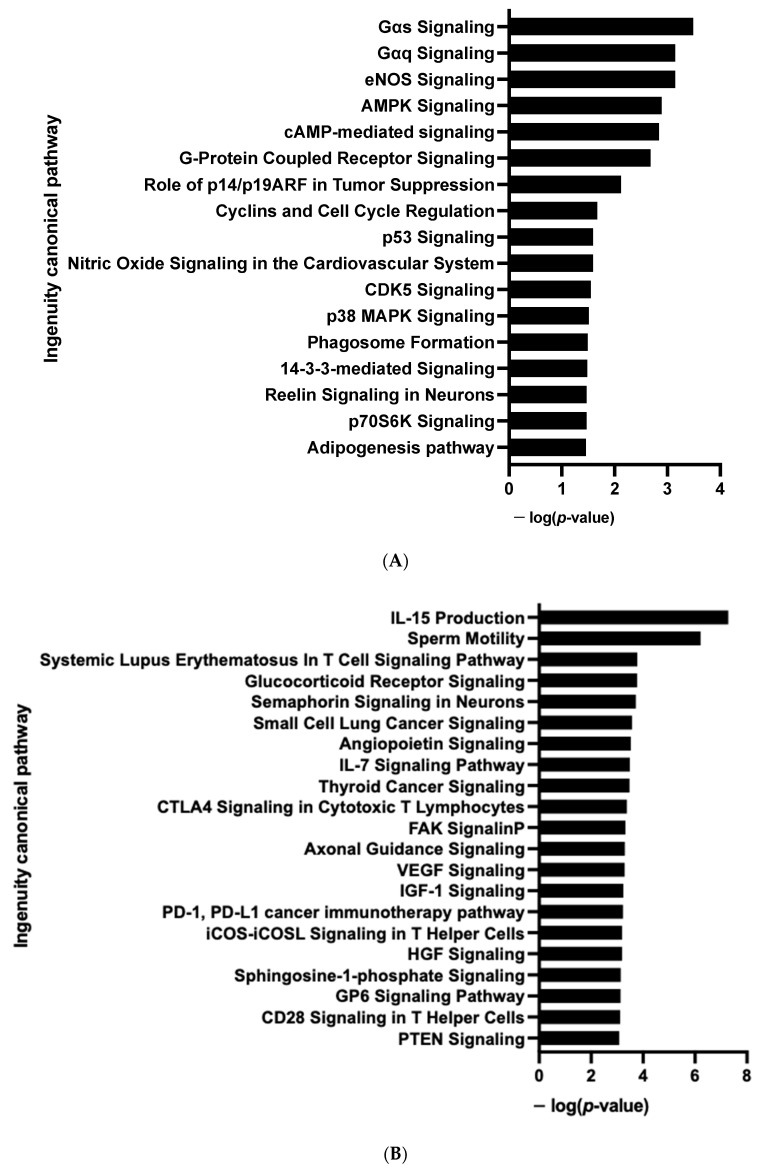
**Enrichment of IPA biological process terms for kinome analysis in 10 days-differentiated (D10) 3D adipospheres following si*CDKN2A* silencing performed at the progenitor stage 3 days before differentiation**. IPA terms are plotted against the negative log of corrected p-values. Most modulated enriched pathways in STK (**A**) and in PTK (**B**).

We then analyzed the effect of the knock-down of *CDKN2A* on the kinome of adipospheres. Significant differences in phosphorylation for 6 out of 144 peptides (STK, Figure 9A and Table S6) and for 8 out of 196 peptides (PTK, Figure 9B and Table S6) were evidenced. Using the Bionavigator analysis to identify potential upstream kinases, we identified several signaling pathways that displayed modified STK (Figure 9C and Table S5) and PTK (Figure 9D and Table S5) phosphorylation. Following combined computional analysis, we observed that, in addition to pro-adipogenic and kinase pathways already highlighted by RNA-seq, AMPK and p38 MAPK which are key players of the browning process [25], are markedly modulated. Differences in phosphorylation linked to the modulation of Gαq- and Gαs-coupled G protein-coupled receptors (GPCR) (STK, Figure 10A) and pro-inflammatory signaling pathways (IL15, IL7) (PTK, Figure 10B) are also modulated [24].

## 4. Discussion

Several studies have pointed out the therapeutic potential of hiPSC-BAP as a promising novel therapy to alleviate the effects of obesity and T2D [18,19]. However, their low capacity for differentiation in brown-like adipocytes in 2D cultures hampers their use for further therapeutic approaches [19]. In recent years, 3D cell culture techniques have received much attention, as these might provide more accurate models of tissues. Indeed, 3D cultures generate changes in lipid accumulation and gene expression, which may lead to a better and closer in vivo differentiation [26]. In order to improve the differentiation capacity of 2D cultures, we developed a novel and more efficient method of using 3D cultures of hiPSC-BAPs, via the formation of an organoid-like structure [19]. Our data confirm previous experiments showing that differentiation into adipospheres improves adipogenesis and browning process capacities compared to conventional monolayer BAP differentiation [16]. 

Fate decisions of multipotent progenitor cells to differentiate into adipocytes are driven by specific signaling pathways. In particular, the adipogenic process occurs in two major phases: commitment to APs and terminal differentiation, which are determined by modified transcriptional, epigenomic and metabolic activities [24]. Here, we showed that the differentiation of hiPSC-BAP from spheroids to adipospheres in a 3D culture results in marked transcriptomic and phosphorylation changes. Comparative transcriptome and kinome analyses of spheroids before differentiation (D0) vs. adipospheres after differentiation (D10) revealed that adipogenesis, oxidative metabolism pathways and browning adipocyte capacity are markedly activated in 10 days-differentiated 3D adipospheres. 

RNA-seq analysis revealed the dynamic expression changes that occur during the commitment of APs toward adipocyte differentiation (i.e., repression of osteoarthritis and hepatic fibrosis pathways). TGFβ and sirtuin signaling pathways, which have emerged as critical anti-adipogenic players, were downregulated. TGFβ 1 and 2 and SIRT1 inhibit PPARγ and CEBPα expression [27,28]. TGF-β 1 inhibition suppresses the proliferation and induces the differentiation of h*iPSC* [19]. By contrast, transcription factor signaling pathways (LXR/RXR, CEBPs and PPARγ), which are the master regulators of adipogenesis [29], were activated. These events are required to promote the growth arrest and differentiation of pre-adipocytes and the progressive expression of a lipogenic transcriptional program (activation of glycolysis, oxidative phosphorylation, fatty acid oxidation and ketogenesis pathways). In line with these findings, the decrease in levels of phosphorylation of MAPK and ERK, JNK, p38 signal-regulated kinases as well as the FGF pathway, which are key regulators of early adipogenic events [24], was evidenced by Pamgene. This might reflect the terminal differentiation of 3D adipospheres into mature adipocytes. In basal 3D culture conditions, the increase in UCP1 [25], IL4 [30] and VEGF [31] expression levels, which is key to the brown adipocyte lineage, suggests that adipocytes already adopt a brown-like phenotype. Indeed, IL-4 enhances the differentiation of APs into committed beige adipogenic precursors [32], and VEGF is synthesized and promotes the angiogenesis in BAT [33]. The activation of cholesterol biosynthesis [34] and angiogenesis (i.e., upregulation of VEGF) [33], and the repression of matrix metalloprotease [35] and hypoxia/inflammation (i.e., dowregulation of HIF1α [36] and acute phase response (APR)) enriched pathways, might reflect an active adipocyte-like remodeling and expansion of the adiposphere. The APR is an early response to inflammation which hampers lipid and glucose utilization in adipocytes [37].

**Figure 11:**
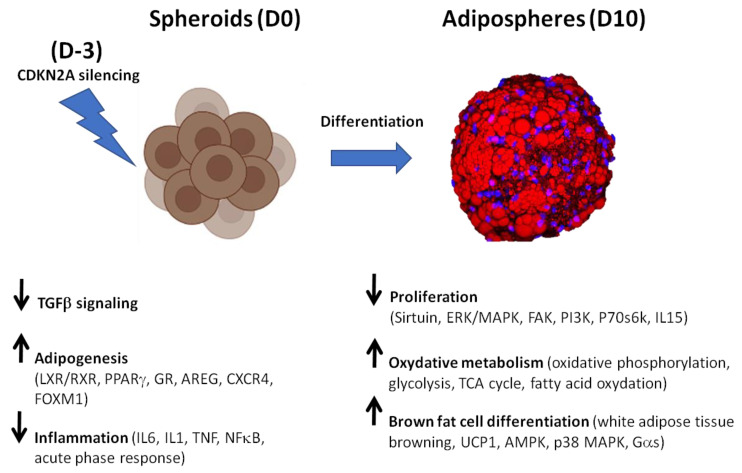
**Effects on silencing *CDKN2A* expression during hiPSC-BAP adipogenic differentiation in a 3D model: from spheroids (D0) to adipospheres (D10)**.

In line with its canonical role in cell-cycle progression and differentiation, the *CDKN2A* locus is well known to promote adipogenesis [15]. It might also be a key determinant of brown adipocyte fate, although underlying mechanisms and cellular pathways remain elusive [13,15]. Thus, we next assessed molecular pathways involved in the browning process in *CDKN2A*-deficient hiPSC-BAPs. *CDKN2A*-deficient spheroids at D0 exhibit greater adipogenic potential with an anti-inflammatory profile (Figure 11). However, no increase in the browning process was evidenced at this stage. In addition to repressed TGFβ and activated adipogenic pathways already highlighted in basal conditions, the most striking observations were the identification of additional modulated signaling pathways, namely the activation of the CXCR4 and the repression of multiple pro-inflammatory signaling pathways. GR and insulin signaling pathways also displayed significant differences in phosphorylation levels. GCs, present in most adipogenic differentiation cocktails, are potent inducers of adipogenesis in vitro. Pre-adipocytes from humans express GR through which GCs stimulate the expression of PPARγ and C/EBPα during adipogenesis [38,39]. Activation of GR decreases pro-inflammatory cytokine expression [40] which is known to inhibit adipogenesis through various pathways, thus constraining the hyperplastic expandability of AT [41]. Insulin is also a powerful inducer of stem cell commitment to adipogenesis via the activation of the PI3K/Akt and MAPK signaling pathways to promote *pro*-adipogenic transcription [42]. CXCR4 promotes proliferation of APs and is required for the acquisition of brown adipocyte features. It also prevents inflammation [43,44].

Strikingly, a marked enrichment of oxidative metabolism and browning process pathways was observed in 10 days-differentiated *CDKN2A*-deficient 3D adipospheres (Figure 11). Although a global increase in adipogenesis and cellular oxidative pathways was already evidenced in basal conditions, the silencing of *CDKN2A* potentiates these pathways along with WAT browning pathways at adiposphere stages of differentiation. The findings of SREBF1 and PPARγ/RXR as major upstream regulators of up-regulated pathways might reflect their dual role in regulating adipogenic and lipogenic pathways [45]. The marked transcriptional activation of AMPK and p38 MAPK and the modulation of the levels of phosphorylation of AMPK and Gα signaling pathways reinforce the idea that adipospheres are fully committed to differentiate into mature brown-like adipocytes [25]. In line with these findings, most of the molecular pathways (i.e., sirtuin, ERK/MAPK, FAK, PI3K, p70S6K) that control pre-adipocyte proliferation [24] were down-regulated, suggesting the terminal differentiation of mature adipocytes (Figure 11). IL15, whose production pathway displays differential phosphorylation, is also known to lower the proliferation rate of pre-adipocytes [46]. On the one hand, AMPK inhibits adipogenesis via blocking the early mitotic clonal expansion. AMPK has a dual role in adipogenesis. AMPK blocks the early mitotic clonal expansion, and later activates the differentiation of pre-adipocytes into mature brown adipocytes [47]. Several studies have reported that AMPK signaling is instrumental in the browning as well as in the energy expenditure of beige adipocytes [48]. Activating intracellular AMPK increases intracellular cAMP and phosphorylates PKA resulting in induced intracellular lipolysis in BAT [48]. p38 MAPK signaling is also a key player in browning [49]. p38 MAPK is a downstream effector kinase of cAMP/PKA signaling in brown adipocytes [50]. During the early phase of adipogenesis, both cAMP and GC signalling pathways promote transcriptional activation, resulting in the commitment of APs to a pre-adipocyte fate and the differentiation of pre-adipocytes [51]. Gαs signaling via GPCRs that activate cAMP/PKA signalling and UCP1-dependent thermogenesis also regulates brown/beige adipocytes [52]. 

One limitation of our study is the lack of functional tests to further investigate the brown fat properties of *CDKN2A*-deficient adipospheres at the cellular level. Additional experiments to compare phenotypic differences in control and *CDKN2A*-deficient adipospheres are also needed to better appreciate whether *CDKN2A* contributes to increased BAT functions and/or morphology. Moreover, at this stage, we cannot rule out that knocking-down *CDKN2A* in hiPSC-BAPs could stimulate cell proliferation. However, no difference in size or cell phenotype was observed microscopically between control and *CDKN2A*-deficient adipospheres throughout the differentiation process and up to 21 days of culture (data not shown). In addition, the RNA-seq data of control and *CDKN2A*-deficient spheroids and adipospheres did not reveal marked modifications in the expression levels of genes involved in signaling pathways that control proliferation. Thus, it suggests that modulating *CDKN2A* expression in hiPSC-BAPs does not lead to uncontrolled proliferation of adipospheres. Given that silencing *CDKN2A* expression in hiPSC-BAPs has limited effect on cell proliferation, it is tempting to speculate that this locus indeed drives alternative pathways from those used for regulating the cell cycle to potentiate the browning process in a 3D system.

Here, we demonstrated that *CDKN2A* plays an important role in brown-like adipogenic recruitment and maturation in a cell-autonomous manner. Our data emphasize the potential effects of this locus in progenitor cells on the browning process, using alternative pathways from those used for regulating the cell cycle. In particular, we showed that AMPK, p38 MAPK and Gαs/cAMP/PKA signaling pathways are key targets of *CDKN2A* silencing (Figure 11). Thus, additional studies are needed to further delineate the contributions of these kinases and to identify both direct and indirect activators underlying the induction of the browning process in *CDKN2A*-deficient stem cells. Thus, targeting alternative *CDKN2A* signaling pathways that may not be involved in tumor suppressive and anti-proliferative effects, but which are driving the browning process in APs, may represent a new strategy to reprogram the cellular response and develop therapeutic approaches against obesity and T2D.

## 5. Conclusions

In conclusion, our results suggest that the *CDKN2A* locus is an important regulator of adipogenesis, oxidative metabolism and the browning process in a cell-autonomous manner.

## Data Availability

Not applicable.

## Supplementary Materials

The following supporting information is available online at https://www.mdpi.com/article/10.3390/cells12060870/s1, Figure S1. Comparative analysis of mRNA expression levels (i.e., Transcripts Per Million (TPM)) between D0 spheroids and D10 adipospheres in 3D hiPSC-BAPs. Figure S2. Upstream regulator analysis of differentially regulated pathways in 10 days-differentiated 3D adipospheres (D10 vs. D0). Figure S3. Gene set enrichment pathway analysis (GSEA)-enrichment plots of representative gene sets from differentially regulated gene expression in 10 days-differentiated 3D adipospheres (D10 vs. D0). Figure S4. Comparative analysis of mRNA expression levels (i.e., Transcripts Per Million (TPM)) between control and *CDKN2A*-deficient D0 spheroids in 3D hiPSC-BAPs. Figure S5. Upstream regulators analysis of differentially regulated pathways between control and *CDKN2A*-deficient D0 spheroids. Figure S6. Gene set enrichment pathway analysis (GSEA)-enrichment plots of representative gene sets from differentially regulated gene expression between control and *CDKN2A*-deficient D0 spheroids. Figure S7. Comparative analysis of mRNA expression levels (i.e., Transcripts Per Million (TPM)) between control and *CDKN2A*-deficient D10 adipospheres in 3D hiPSC-BAPs. Figure S8. Upstream regulator analysis of differentially regulated pathways between control and *CDKN2A*-deficient D10 adipospheres. Figure S9. Gene set enrichment pathway analysis (GSEA)-enrichment plots of representative gene sets from differentially regulated gene expression between control and *CDKN2A*-deficient D10 adipospheres. Table S1. Kinases score table (PTK and STK) D0 spheroids vs. D10 adipospheres. Table S2. Peptides score table (PTK and STK) D0 spheroids vs. D10 adipospheres. Table S3. Kinases score table (PTK and STK) control vs *CDKN2A*-deficient D0 spheroids. Table S4. Peptides score table (PTK and STK) control vs. *CDKN2A*-deficient D0 spheroids. Table S5. Kinases score table (PTK and STK) control vs. *CDKN2A*-deficient D10 adipospheres. Table S6. Peptides score table (PTK and STK) control vs. *CDKN2A*-deficient D10 adipospheres.

## References

[1] Qasim A., Turcotte M., de Souza R.J., Samaan M.C., Champredon D., Dushoff J., Speakman J.R., Meyre D. (2018). On the Origin of Obesity: Identifying the Biological, Environmental and Cultural Drivers of Genetic Risk among Human Populations. Obes. Rev..

[2] Goodpaster B.H., Sparks L.M. (2017). Metabolic Flexibility in Health and Disease. Cell Metab..

[3] Herz C.T., Kiefer F.W. (2019). Adipose Tissue Browning in Mice and Humans. J. Endocrinol..

[4] Kajimura S., Spiegelman B.M., Seale P. (2015). Brown and Beige Fat: Physiological Roles beyond Heat Generation. Cell Metab..

[5] Wu J., Boström P., Sparks L.M., Ye L., Choi J.H., Giang A.-H., Khandekar M., Virtanen K.A., Nuutila P., Schaart G. (2012). Beige Adipocytes Are a Distinct Type of Thermogenic Fat Cell in Mouse and Human. Cell.

[6] Hanssen M.J.W., Hoeks J., Brans B., van der Lans A.A.J.J., Schaart G., van den Driessche J.J., Jörgensen J.A., Boekschoten M.V., Hesselink M.K.C., Havekes B. (2015). Short-Term Cold Acclimation Improves Insulin Sensitivity in Patients with Type 2 Diabetes Mellitus. Nat. Med..

[7] White J.D., Dewal R.S., Stanford K.I. (2019). The Beneficial Effects of Brown Adipose Tissue Transplantation. Mol. Aspects Med..

[8] Hepler C., Vishvanath L., Gupta R.K. (2017). Sorting out Adipocyte Precursors and Their Role in Physiology and Disease. Genes Dev..

[9] Berry R., Rodeheffer M.S. (2013). Characterization of the Adipocyte Cellular Lineage in Vivo. Nat. Cell Biol..

[10] Gao H., Volat F., Sandhow L., Galitzky J., Nguyen T., Esteve D., Åström G., Mejhert N., Ledoux S., Thalamas C. (2017). CD36 Is a Marker of Human Adipocyte Progenitors with Pronounced Adipogenic and Triglyceride Accumulation Potential. Stem Cells.

[11] Hannou S.A., Wouters K., Paumelle R., Staels B. (2015). Functional Genomics of the CDKN2A/B Locus in Cardiovascular and Metabolic Disease: What Have We Learned from GWASs?. Trends Endocrinol. Metab..

[12] Morris A.P., Voight B.F., Teslovich T.M., Ferreira T., Segrè A.V., Steinthorsdottir V., Strawbridge R.J., Khan H., Grallert H., Mahajan A. (2012). Large-Scale Association Analysis Provides Insights into the Genetic Architecture and Pathophysiology of Type 2 Diabetes. Nat. Genet..

[13] Berry D.C., Jiang Y., Arpke R.W., Close E.L., Uchida A., Reading D., Berglund E.D., Kyba M., Graff J.M. (2017). Cellular Aging Contributes to Failure of Cold-Induced Beige Adipocyte Formation in Old Mice and Humans. Cell Metab..

[14] Svensson P.-A., Wahlstrand B., Olsson M., Froguel P., Falchi M., Bergman R.N., McTernan P.G., Hedner T., Carlsson L.M.S., Jacobson P. (2014). CDKN2B Expression and Subcutaneous Adipose Tissue Expandability: Possible Influence of the 9p21 Atherosclerosis Locus. Biochem. Biophys. Res. Commun..

[15] Kahoul Y., Oger F., Montaigne J., Froguel P., Breton C., Annicotte J.-S. (2020). Emerging Roles for the INK4a/ARF (CDKN2A) Locus in Adipose Tissue: Implications for Obesity and Type 2 Diabetes. Biomolecules.

[16] Rabhi N., Hannou S.A., Gromada X., Salas E., Yao X., Oger F., Carney C., Lopez-Mejia I.C., Durand E., Rabearivelo I. (2018). Cdkn2a Deficiency Promotes Adipose Tissue Browning. Mol. Metab..

[17] Park J., Shin S., Liu L., Jahan I., Ong S.-G., Xu P., Berry D.C., Jiang Y. (2021). Progenitor-like Characteristics in a Subgroup of UCP1+ Cells within White Adipose Tissue. Dev. Cell.

[18] Hafner A.-L., Contet J., Ravaud C., Yao X., Villageois P., Suknuntha K., Annab K., Peraldi P., Binetruy B., Slukvin I.I. (2016). Brown-like Adipose Progenitors Derived from Human Induced Pluripotent Stem Cells: Identification of Critical Pathways Governing Their Adipogenic Capacity. Sci. Rep..

[19] Yao X., Dani V., Dani C. (2020). Human Pluripotent Stem Cells: A Relevant Model to Identify Pathways Governing Thermogenic Adipocyte Generation. Front. Endocrinol..

[20] Mohsen-Kanson T., Hafner A.-L., Wdziekonski B., Takashima Y., Villageois P., Carrière A., Svensson M., Bagnis C., Chignon-Sicard B., Svensson P.-A. (2014). Differentiation of Human Induced Pluripotent Stem Cells into Brown and White Adipocytes: Role of Pax3. Stem Cells.

[21] Yao X., Dani C. (2022). A Simple Method for Generating, Clearing, and Imaging Pre-Vascularized 3D Adipospheres Derived from Human IPS Cells. Methods Mol. Biol..

[22] Bianco A.C., Salvatore D., Gereben B., Berry M.J., Larsen P.R. (2002). Biochemistry, Cellular and Molecular Biology, and Physiological Roles of the Iodothyronine Selenodeiodinases. Endocr. Rev..

[23] Wagner M.S., Wajner S.M., Dora J.M., Maia A.L. (2007). Regulation of Dio2 Gene Expression by Thyroid Hormones in Normal and Type 1 Deiodinase-Deficient C3H Mice. J. Endocrinol..

[24] Ambele M.A., Dhanraj P., Giles R., Pepper M.S. (2020). Adipogenesis: A Complex Interplay of Multiple Molecular Determinants and Pathways. Int. J. Mol. Sci..

[25] Machado S.A., Pasquarelli-do-Nascimento G., da Silva D.S., Farias G.R., de Oliveira Santos I., Baptista L.B., Magalhães K.G. (2022). Browning of the White Adipose Tissue Regulation: New Insights into Nutritional and Metabolic Relevance in Health and Diseases. Nutr. Metab..

[26] Shen J.X., Couchet M., Dufau J., de Castro Barbosa T., Ulbrich M.H., Helmstädter M., Kemas A.M., Zandi Shafagh R., Marques M.-A., Hansen J.B. (2021). 3D Adipose Tissue Culture Links the Organotypic Microenvironment to Improved Adipogenesis. Adv. Sci..

[27] Ahdjoudj S., Kaabeche K., Holy X., Fromigué O., Modrowski D., Zérath E., Marie P.J. (2005). Transforming Growth Factor-Beta Inhibits CCAAT/Enhancer-Binding Protein Expression and PPARgamma Activity in Unloaded Bone Marrow Stromal Cells. Exp. Cell Res..

[28] Majeed Y., Halabi N., Madani A.Y., Engelke R., Bhagwat A.M., Abdesselem H., Agha M.V., Vakayil M., Courjaret R., Goswami N. (2021). SIRT1 Promotes Lipid Metabolism and Mitochondrial Biogenesis in Adipocytes and Coordinates Adipogenesis by Targeting Key Enzymatic Pathways. Sci. Rep..

[29] Lefterova M.I., Haakonsson A.K., Lazar M.A., Mandrup S. (2014). PPARγ and the Global Map of Adipogenesis and Beyond. Trends Endocrinol. Metab..

[30] Su S., Guntur A.R., Nguyen D.C., Fakory S.S., Doucette C.C., Leech C., Lotana H., Kelley M., Kohli J., Martino J. (2018). A Renewable Source of Human Beige Adipocytes for Development of Therapies to Treat Metabolic Syndrome. Cell Rep..

[31] Nishio M., Yoneshiro T., Nakahara M., Suzuki S., Saeki K., Hasegawa M., Kawai Y., Akutsu H., Umezawa A., Yasuda K. (2012). Production of Functional Classical Brown Adipocytes from Human Pluripotent Stem Cells Using Specific Hemopoietin Cocktail without Gene Transfer. Cell Metab..

[32] Lizcano F., Vargas D., Gómez Á., Torrado A. (2017). Human ADMC-Derived Adipocyte Thermogenic Capacity Is Regulated by IL-4 Receptor. Stem Cells Int..

[33] Jin H., Li D., Wang X., Jia J., Chen Y., Yao Y., Zhao C., Lu X., Zhang S., Togo J. (2018). VEGF and VEGFB Play Balancing Roles in Adipose Differentiation, Gene Expression, and Function. Endocrinology.

[34] Chung S., Parks J.S. (2016). Dietary Cholesterol Effects on Adipose Tissue Inflammation. Curr. Opin. Lipidol..

[35] Chavey C., Mari B., Monthouel M.-N., Bonnafous S., Anglard P., Van Obberghen E., Tartare-Deckert S. (2003). Matrix Metalloproteinases Are Differentially Expressed in Adipose Tissue during Obesity and Modulate Adipocyte Differentiation. J. Biol. Chem..

[36] Wagegg M., Gaber T., Lohanatha F.L., Hahne M., Strehl C., Fangradt M., Tran C.L., Schönbeck K., Hoff P., Ode A. (2012). Hypoxia Promotes Osteogenesis but Suppresses Adipogenesis of Human Mesenchymal Stromal Cells in a Hypoxia-Inducible Factor-1 Dependent Manner. PLoS ONE.

[37] Venteclef N., Jakobsson T., Steffensen K.R., Treuter E. (2011). Metabolic Nuclear Receptor Signaling and the Inflammatory Acute Phase Response. Trends Endocrinol. Metab..

[38] Wu Z., Bucher N.L., Farmer S.R. (1996). Induction of Peroxisome Proliferator-Activated Receptor Gamma during the Conversion of 3T3 Fibroblasts into Adipocytes Is Mediated by C/EBPbeta, C/EBPdelta, and Glucocorticoids. Mol. Cell Biol..

[39] Smas C.M., Chen L., Zhao L., Latasa M.J., Sul H.S. (1999). Transcriptional Repression of Pref-1 by Glucocorticoids Promotes 3T3-L1 Adipocyte Differentiation. J. Biol. Chem..

[40] Desarzens S., Faresse N. (2016). Adipocyte Glucocorticoid Receptor Has a Minor Contribution in Adipose Tissue Growth. J. Endocrinol..

[41] Jiang N., Li Y., Shu T., Wang J. (2019). Cytokines and Inflammation in Adipogenesis: An Updated Review. Front. Med.

[42] Cignarelli A., Genchi V.A., Perrini S., Natalicchio A., Laviola L., Giorgino F. (2019). Insulin and Insulin Receptors in Adipose Tissue Development. Int. J. Mol. Sci..

[43] van den Berg S.M., van Dam A.D., Rensen P.C.N., de Winther M.P.J., Lutgens E. (2017). Immune Modulation of Brown(Ing) Adipose Tissue in Obesity. Endocr. Rev..

[44] Kurita K., Ishikawa K., Takeda K., Fujimoto M., Ono H., Kumagai J., Inoue H., Yokoh H., Yokote K. (2019). CXCL12-CXCR4 Pathway Activates Brown Adipocytes and Induces Insulin Resistance in CXCR4-Deficient Mice under High-Fat Diet. Sci. Rep..

[45] Ahmadian M., Suh J.M., Hah N., Liddle C., Atkins A.R., Downes M., Evans R.M. (2013). PPARγ Signaling and Metabolism: The Good, the Bad and the Future. Nat. Med..

[46] Fuster G., Almendro V., Fontes-Oliveira C.C., Toledo M., Costelli P., Busquets S., López-Soriano F.J., Argilés J.M. (2011). Interleukin-15 Affects Differentiation and Apoptosis in Adipocytes: Implications in Obesity. Lipids.

[47] Ahmad B., Serpell C.J., Fong I.L., Wong E.H. (2020). Molecular Mechanisms of Adipogenesis: The Anti-Adipogenic Role of AMP-Activated Protein Kinase. Front. Mol. Biosci..

[48] van der Vaart J.I., Boon M.R., Houtkooper R.H. (2021). The Role of AMPK Signaling in Brown Adipose Tissue Activation. Cells.

[49] Leiva M., Matesanz N., Pulgarín-Alfaro M., Nikolic I., Sabio G. (2020). Uncovering the Role of P38 Family Members in Adipose Tissue Physiology. Front. Endocrinol..

[50] Bordicchia M., Liu D., Amri E.-Z., Ailhaud G., Dessì-Fulgheri P., Zhang C., Takahashi N., Sarzani R., Collins S. (2012). Cardiac Natriuretic Peptides Act via P38 MAPK to Induce the Brown Fat Thermogenic Program in Mouse and Human Adipocytes. J. Clin. Investig..

[51] Petersen R.K., Madsen L., Pedersen L.M., Hallenborg P., Hagland H., Viste K., Døskeland S.O., Kristiansen K. (2008). Cyclic AMP (CAMP)-Mediated Stimulation of Adipocyte Differentiation Requires the Synergistic Action of Epac- and CAMP-Dependent Protein Kinase-Dependent Processes. Mol. Cell. Biol..

[52] Klepac K., Kilić A., Gnad T., Brown L.M., Herrmann B., Wilderman A., Balkow A., Glöde A., Simon K., Lidell M.E. (2016). The Gq Signalling Pathway Inhibits Brown and Beige Adipose Tissue. Nat. Commun..

