# Supplementary material for: Knocking Down CDKN2A in 3D hiPSC-Derived Brown Adipose Progenitors Potentiates Differentiation, Oxidative Metabolism and Browning Process
Source: Cells. 2023 Mar 10;12(6):870. doi: 10.3390/cells12060870 (PMC10047013; doi:10.3390/cells12060870)
Supplement: Supplementary file 1 [file cells-12-00870-s001.zip › cells-2222475-supplementary.pdf]

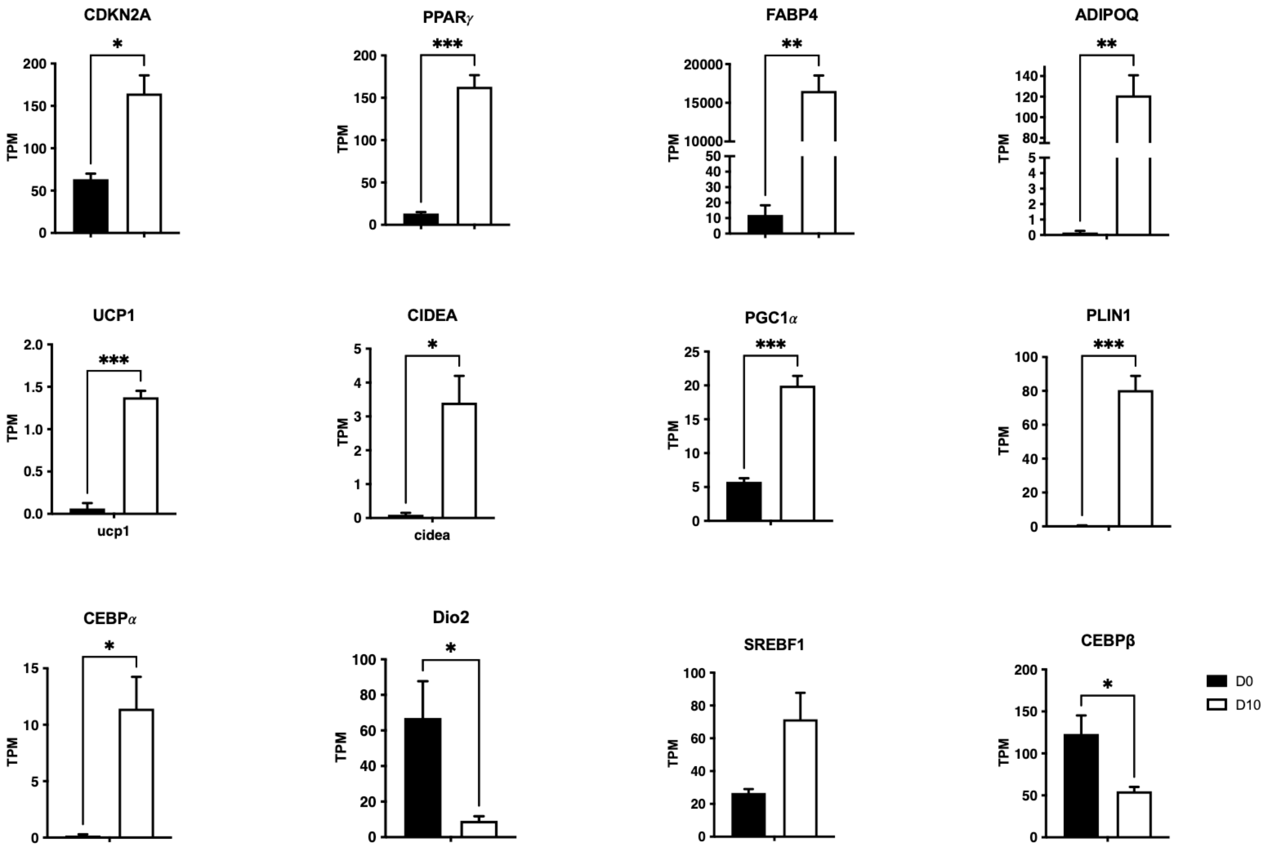

Figure S1. Comparative analysis of mRNA expression levels (i.e., Transcripts Per Million (TPM)) between D0 spheroids and D10 adipospheres in 3D hiPSC-BAPs.

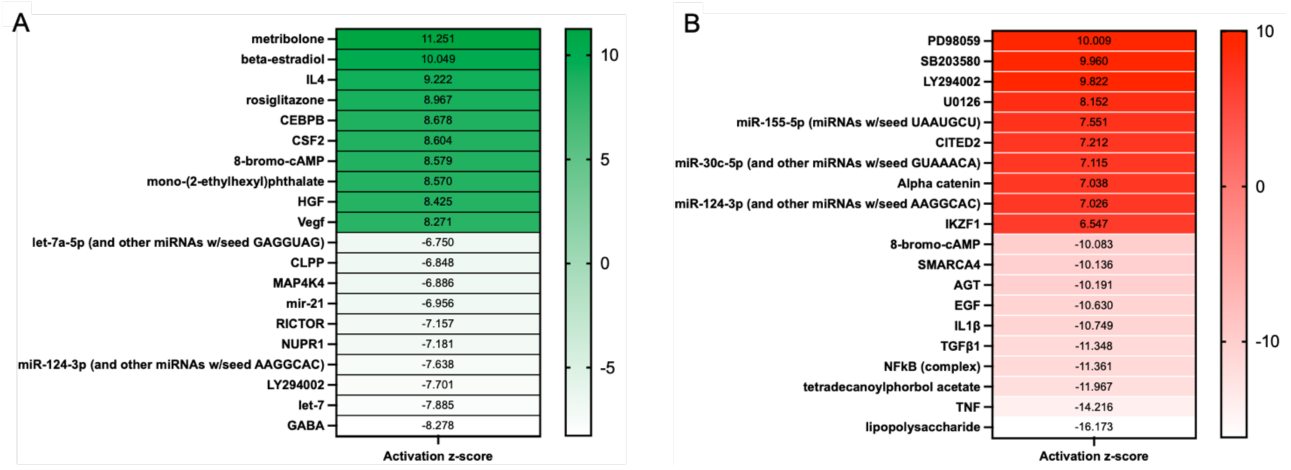

Figure S2. Upstream regulator analysis of differentially regulated pathways in 10 days-differentiated 3D adipospheres (D10 *vs* D0). (A) Upstream regulators of up-regulated pathways. (B) Upstream regulators of down-regulated pathways.

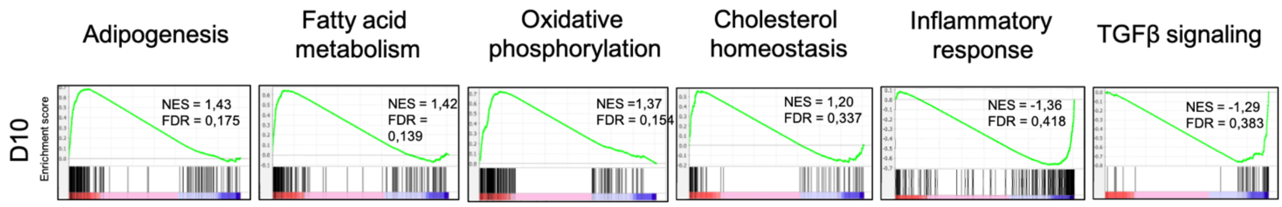

Figure S3. Gene set enrichment pathway analysis (GSEA)-enrichment plots of representative gene sets from differentially regulated gene expression in 10 days-differentiated 3D adipospheres (D10 *vs* D0).

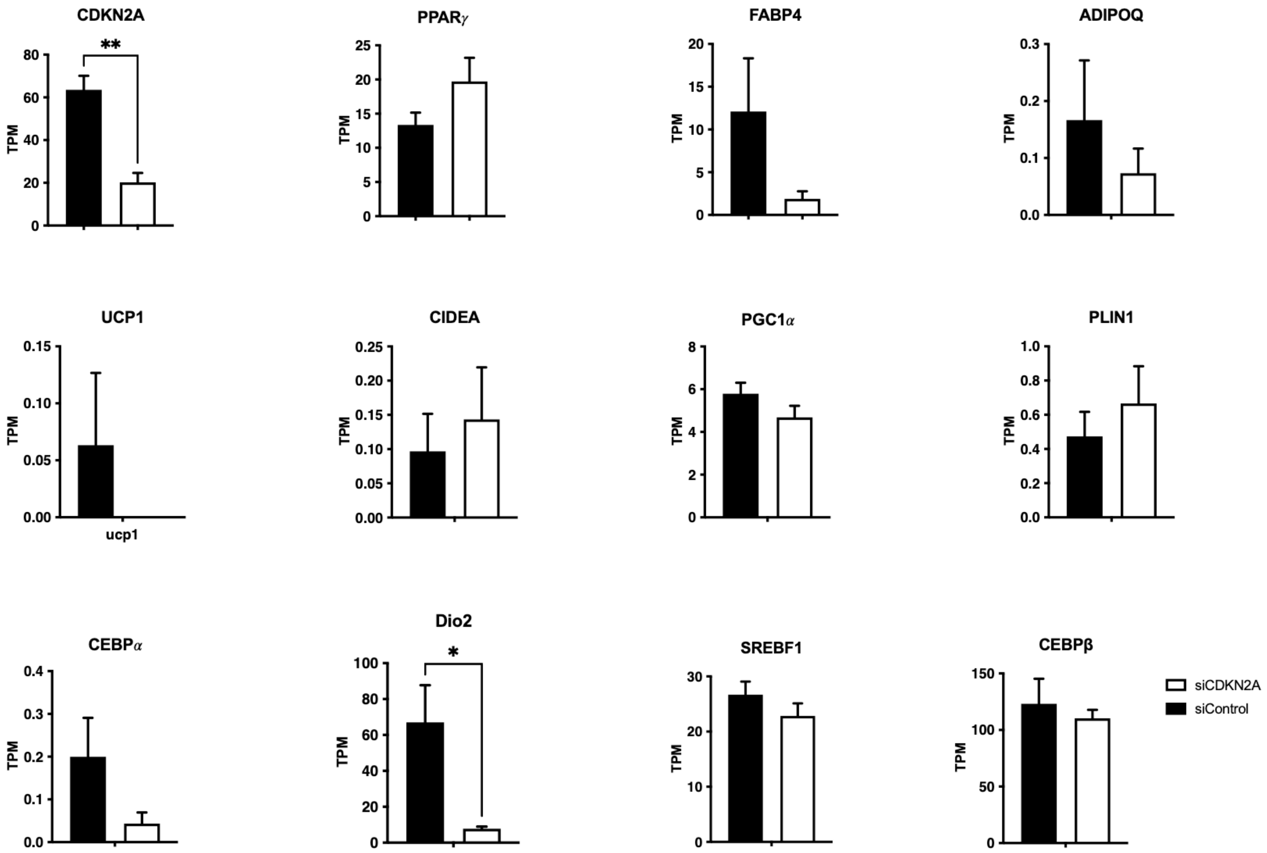

Figure S4. Comparative analysis of mRNA expression levels (i.e., Transcripts Per Million (TPM)) between control and CDKN2A-deficient D0 spheroids in 3D hiPSC-BAPs.

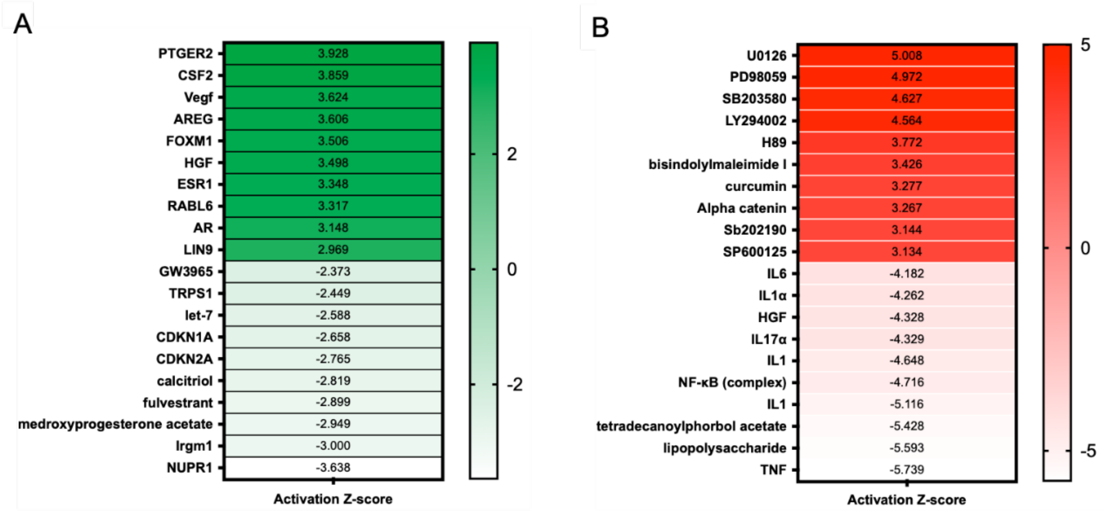

Figure S5. Upstream regulators analysis of differentially regulated pathways between control and CDKN2A-deficient D0 spheroids. (A) Upstream regulators of up-regulated pathways. (B) Upstream regulators of down-regulated pathways.

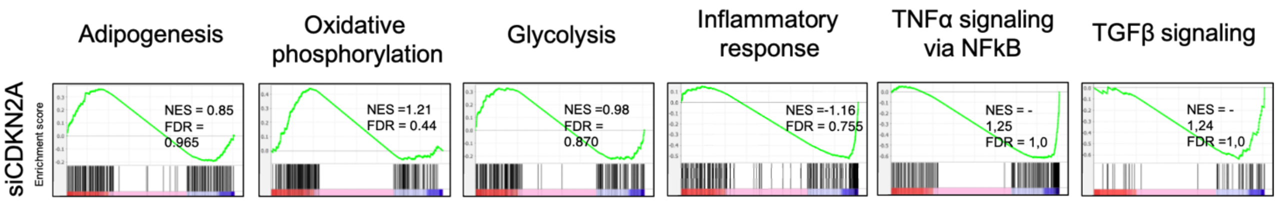

Figure S6. Gene set enrichment pathway analysis (GSEA)-enrichment plots of representative gene sets from differentially regulated gene expression between control and CDKN2A-deficient D0 spheroids.

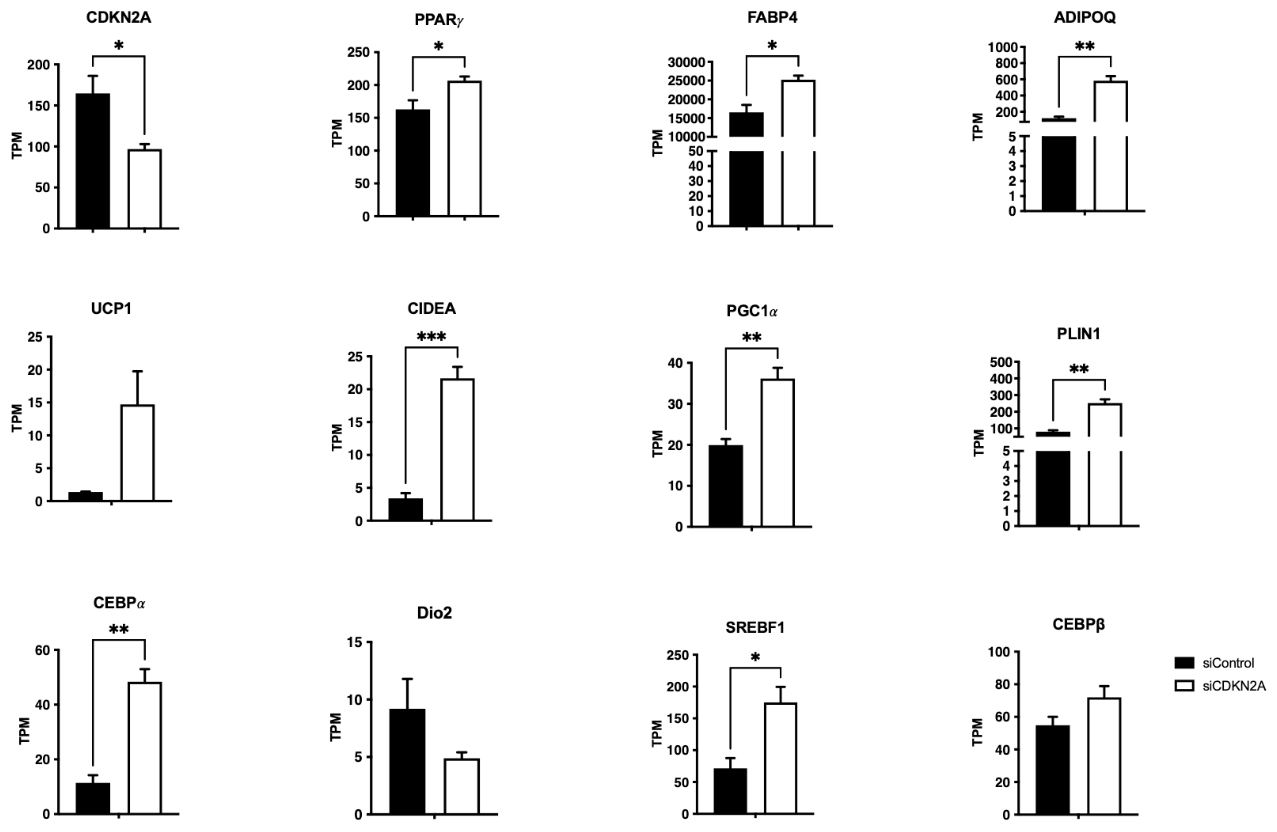

Figure S7. Comparative analysis of mRNA expression levels (i.e., Transcripts Per Million (TPM)) between control and CDKN2A-deficient D10 adipospheres in 3D hiPSC-BAPs.

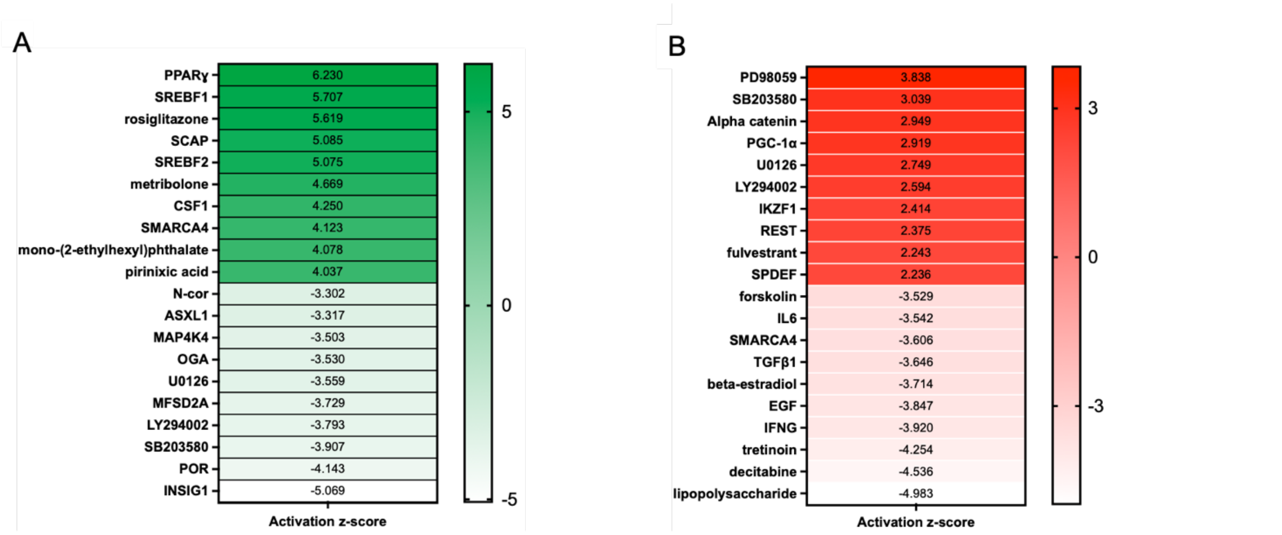

Figure S8. Upstream regulator analysis of differentially regulated pathways between control and CDKN2A-deficient D10 adipospheres. (A) Upstream regulators of up-regulated pathways. (B) Upstream regulators of down-regulated pathways.

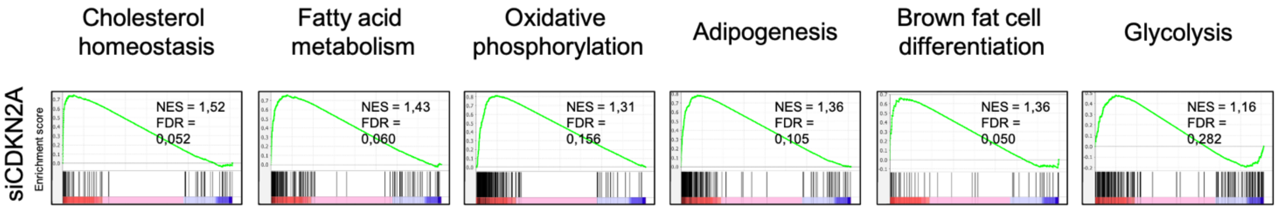

Figure S9. Gene set enrichment pathway analysis (GSEA)-enrichment plots of representative gene sets from differentially regulated gene expression between control and CDKN2A-deficient D10 adiospheres.

Rank	PTK Kinase Name	Mean Final Score	Mean Kinase Statistic
1	BLK	2,587897505	-3,851509751
2	FGFR1	2,434166938	-4,729220701
3	Lck	2,276231552	-3,122426279
4	FGFR4	2,192615108	-3,741560736
5	FGFR2	2,161490566	-3,902745721
6	FGFR3	2,129061326	-3,938490642
7	Yes	2,114281846	-3,048435562
8	Arg	2,111499894	-2,817203637
9	HCK	2,106587086	-2,853751317
10	Syk	2,068883214	-2,49305365
11	FmS/CSFR	2,021075341	-4,428963
12	Src	1,994184101	-2,767348433
13	Ret	1,834551184	-3,046858301
14	HER4	1,775512349	-3,083931408
15	KDR	1,769444569	-2,993196455
16	Abl	1,75558178	-2,483894014
17	HER2	1,727397176	-2,78107761
18	EphA4	1,721673581	-2,700252455
19	Brk	1,70960674	-2,453540913
20	TRKC	1,68861498	-3,001516888
21	ZAP70	1,680060954	-2,412086269
22	TRKB	1,675522302	-2,863176951
23	FLT4	1,671157552	-2,898290776
24	Fer	1,6622124	-2,911329189
25	EphA2	1,660536394	-2,576904562
26	ALK	1,651085962	-2,431505901
27	Axl	1,613139284	-2,429260085
28	TXK	1,588563821	-2,275540567
29	Mer	1,535486364	-2,349181745
30	TRKA	1,493395938	-2,389443881
31	HER3	1,484588524	-2,355913006
32	EphA8	1,434319005	-1,990553879
33	Kit	1,406791102	-2,165697741
34	Fyn	1,395468092	-2,081310579
35	FAK1	1,366390088	-2,160388369
36	EphA1	1,363065651	-1,878133766
37	TEC	1,343937478	-2,091579999
38	Met	1,339364338	-2,125280344
39	FAK2	1,313622961	-2,108979832
40	EphA5	1,313363731	-1,747545347
41	EGFR	1,304298011	-2,14383432

Rank	STK Kinase Name	Mean Final Score	Mean Kinase Statistic
1	ERK1	3,440334482	-0,863973328
2	ERK2	3,390530996	-0,823225582
3	CDK2	2,777646678	-0,697106888
4	ERK5	2,71681798	-0,795082149
5	CDK4	2,654324419	-0,876872073
6	AlphaK1	2,649055128	-1,206198271
7	CDK6	2,534908645	-0,904526514
8	CDK11	2,510755801	-1,034229499
9	CDKL5	2,497973893	-0,995168141
10	p38[gamma]	2,414115588	-0,818466189
11	p38[beta]	2,369713449	-0,907410647
12	p38[delta]	2,344169093	-0,656678685
13	CDK7	2,253681934	-0,66645641
14	ERK7	2,242300375	-1,013376722
15	JNK1	2,128848493	-0,589416351
16	JNK3	2,08478833	-0,589779677
17	DAPK3	2,082599532	-0,964958792
18	MAPK14	1,885650254	-0,591183929
19	JNK2	1,884196885	-0,605353358
20	ICK	1,867484165	-0,807577604
21	CDC2/CDK1	1,840782433	-0,593473044
22	CDK3	1,760787807	-0,583485707
23	CDK5	1,7486884	-0,575221591
24	TBK1	1,582086776	-0,730407142
25	CDKL1	1,513518422	-0,57495913
26	PAK1	1,503421862	-0,701768133
27	NuaK1	1,499836525	-0,570182551
28	CDKL2	1,447380447	-0,683231764
29	CDK9	1,376068794	-0,414469919

Table S1. Kinases score table (PTK and STK) of D0 spheroids *vs* D10 adipospheres.

Peptides	STK/PTK	LFC	Pvalue
ARAF_297_307	PTK	-0,6134956	8,50E-04
PDCD1_221_229_FS219_220KK	PTK	-1,133137	2,70E-03
VGFR3_1061_1073	PTK	-1,6498542	7,12E-03
PTN11_57_67	PTK	-0,9472599	7,42E-03
AKT1_309_321_C310S	PTK	-0,624779	8,26E-03
AKT1_320_332	PTK	-1,6804954	9,42E-03
MK10_216_228	PTK	-0,5112407	1,42E-02
RAF1_332_344	PTK	-0,5062263	1,75E-02
PRRX2_202_214	PTK	-0,5016437	2,43E-02
BTLA_252_262	PTK	-0,6638995	2,95E-02
MK12_180_189_M182B	PTK	-0,3855168	3,59E-02
TYK2_1048_1060	PTK	-0,3830287	3,79E-02
ZAP70_485_497	PTK	-0,8363056	4,16E-02
FGFR1_761_773	PTK	-2,0622191	4,95E-02
RB_242_254	STK	2,04E-02	5,40E-03
RAP1B_172_184	STK	-0,5611579	2,41E-02
CD27_212_224	STK	-0,2998864	3,85E-02
FRAP_2443_2455	STK	-0,1546803	4,01E-02
ADDB_706_718	STK	0,10405827	4,26E-02

Table S2. Peptides score table (PTK and STK) of D0 spheroids *vs* D10 adipospheres.

Rank	PTK Kinase Name	Mean Final Score	Mean Kinase Statistic
1	HCK	1,961220128	0,460651987
2	FGFR1	1,86926031	0,562869443
3	Src	1,472683358	0,29411269
4	KDR	1,391021789	0,374796464
5	CSK	1,328089579	0,289891645
6	FGFR3	1,315730624	0,452245082
7	FGFR4	1,311057037	0,408545378
8	TXK	1,305227154	0,327402154
Rank	STK Kinase Name	Mean Final Score	Mean Kinase Statistic
1	DAPK2	1,898140805	0,449021666
2	Akt1/PKB[alpha]	1,656808643	0,229981306
3	PKC[theta]	1,653460552	0,243450872
4	GSK3[alpha]	1,628074666	0,391124939
5	PKC[alpha]	1,593043453	0,223456938
6	PKC[delta]	1,537349512	0,248610717
7	AMPK[alpha]1	1,512322373	0,305743225
8	CHK1	1,444867338	0,302709376
9	Akt2/PKB[beta]	1,436188077	0,222417622
10	MSK2	1,414384635	0,357649245
11	GSK3[beta]	1,355080135	0,3225343
12	PKC[zeta]	1,34879529	0,275297291
13	PKN1/PRK1	1,330140997	0,279499594

Table S3. Kinases score table (PTK and STK) of control *vs* CDKN2A-deficient D0 spheroids.

Peptides	STK/PTK	LFC	Pvalue
PDPK1_369_381	PTK	-5,27E-02	3,80E-03
CRK_214_226	PTK	0,7016654	7,52E-03
CDK1_9_21	PTK	-0,3317584	1,28E-02
FER_707_719	PTK	0,17346191	2,10E-02
PRRX2_202_214	PTK	0,39735985	2,43E-02
ZAP70_485_497	PTK	0,30168295	4,22E-02
AKT1_309_321_C310S	PTK	0,34416914	4,36E-02
FRK_380_392	PTK	-0,1401911	4,60E-02
FES_706_718	PTK	0,15176122	4,71E-02
PTN6_531_541	PTK	-0,2107744	4,89E-02
ANXA1_209_221	STK	-0,5602635	2,80E-02

Table S4. Peptides score table (PTK and STK) of control *vs* CDKN2A-deficient D0 spheroids.

Rank	PTK Kinase Nar	Mean Final Score	Mean Kinase Statistic
1	Syk	2,294361873	0,564046227
2	HCK	1,890351743	0,662544356
3	Yes	1,864642748	0,683415043
4	FAK2	1,863042834	0,66094871
5	FAK1	1,771613272	0,575407491
6	CTK	1,675012084	0,65030287
7	EphA2	1,591901866	0,923746565
8	EphA4	1,576631078	0,951222036
9	Lyn	1,549545902	0,639951533
10	Lck	1,439150011	0,580564358
11	Src	1,330285568	0,552129477

Rank	STK Kinase Name	Mean Final Score	Mean Kinase Statistic
1	ERK2	3,423870314	0,700972037
2	ERK1	3,205055855	0,677369966
3	CDK2	3,072718153	0,609376463
4	ERK5	2,348087965	0,60119793
5	PAK1	2,311870131	0,725189969
6	CDK11	2,291219104	0,805662721
7	JNK1	2,221518702	0,501217777
8	JNK3	2,206681046	0,50144175
9	JNK2	2,106309256	0,519506202
10	p38[delta]	2,075594885	0,526564831
11	DAPK3	2,07139934	0,855769447
12	RSK2	2,00884815	0,617379413
13	p38[beta]	1,993876061	0,654970143
14	p38[gamma]	1,940728697	0,600072794
15	CDK3	1,894011488	0,5366334
16	PKN1/PRK1	1,856057022	0,600441074
17	CDK7	1,759290209	0,531977845
18	MSK1	1,73670375	0,586032711
19	CDC2/CDK1	1,697895248	0,519995668
20	BRAF	1,616211468	0,628644218
21	CDK6	1,558737821	0,585599421
22	ARAF	1,539487347	0,626322331
23	RSKL1	1,488029394	0,592039418
24	CDKL2	1,459770662	0,616678444
25	CDK9	1,450168809	0,550907484
26	MAPK14	1,440425219	0,492400418
27	CDK4	1,368212912	0,544788554

Table S5. Kinases score table (PTK and STK) of control *vs* CDKN2A-deficient D10 adipospheres.

Peptides	STK/PTK	LFC	Pvalue
RON_1346_1358	PTK	0,67521787	1,49E-03
CD3Z_135_147	PTK	0,57993567	5,52E-03
RET_1022_1034	PTK	0,11388135	1,21E-02
ZBT16_621_633	PTK	0,69783235	2,09E-02
AKT1_320_332	PTK	0,18023205	2,25E-02
FES_706_718	PTK	0,15820622	2,74E-02
FAK1_569_581	PTK	3,58E-02	2,82E-02
K2C6B_53_65	PTK	-0,9405434	3,75E-02
TAU_524_536	STK	0,51969433	1,70E-02
ACM1_444_456	STK	0,25251928	3,12E-02
RB_242_254	STK	0,16459639	3,42E-02
MARCS_160_172	STK	0,35433802	3,75E-02
ACM5_494_506	STK	0,23127301	4,34E-02
GSUB_61_73	STK	0,37231508	4,96E-02

Table S6. Peptides score table (PTK and STK) of control *vs* CDKN2A-deficient D10 adipospheres.
